# Coupled chemistry kinetics demonstrate the utility of functionalized Sup35 amyloid nanofibrils in biocatalytic cascades

**DOI:** 10.1074/jbc.RA119.008455

**Published:** 2019-08-15

**Authors:** Benjamin Schmuck, Mikael Gudmundsson, Torleif Härd, Mats Sandgren

**Affiliations:** Department of Molecular Sciences, Swedish University of Agricultural Sciences, Box 7015, 750 07 Uppsala, Sweden

**Keywords:** amyloid, enzyme kinetics, fusion protein, protein aggregation, protein chimera, protein engineering, aldose sugar dehydrogenase, beta-xylosidase, protein nanofibrils, xylan, xylanase

## Abstract

Concerns over the environment are a central driver for designing cell-free enzymatic cascade reactions that synthesize non–petrol-based commodity compounds. An often-suggested strategy that would demonstrate the economic competitiveness of this technology is recycling of valuable enzymes through their immobilization. For this purpose, amyloid nanofibrils are an ideal scaffold to realize chemistry-free covalent enzyme immobilization on a material that offers a large surface area. However, in most instances, only single enzyme–functionalized amyloid fibrils have so far been studied. To embark on the next stage, here we displayed xylanase A, β-xylosidase, and an aldose sugar dehydrogenase on Sup35(1–61) nanofibrils to convert beechwood xylan to xylonolactone. We characterized this enzymatic cascade by measuring the time-dependent accumulation of xylose, xylooligomers, and xylonolactone. Furthermore, we studied the effects of relative enzyme concentrations, pH, temperature, and agitation on product formation. Our investigations revealed that a modular cascade with a mixture of xylanase and β-xylosidase, followed by product removal and separate oxidation of xylose with the aldose sugar dehydrogenase, is more productive than an enzyme mix containing all of these enzymes together. Moreover, we found that the nanofibril-coupled enzymes do not lose activity compared with their native state. These findings provide proof of concept of the feasibility of functionalized Sup35(1–61) fibrils as a molecular scaffold for biocatalytic cascades consisting of reusable enzymes that can be used in biotechnology.

## Introduction

Enzymes are nature's renewable and biodegradable catalysts. A part of a cell's efficient utilization of enzymes for metabolic purposes is accomplished via organization and compartmentalization of these biocatalysts (1). The immobilization of enzymes occurs intracellularly in organelles, membranes, and the cytoskeleton, as well as extracellularly on cell walls, such as the flagella (2) or cellulosomes (3).

Inspired by these naturally existing biocatalytic cascades, we aimed to explore an efficient and sustainable synthesis pathway for the production of compounds that otherwise have petrochemical origins (4–7). An option to realize specific chemical cascade reactions is through genetic engineering of microorganisms. The rationale behind microorganism engineering is to exploit already existing biosynthetic pathways, which are modified to produce the target chemicals. However, this can be a time-consuming task that requires hundreds of accumulated working years and is often limited by our understanding of the organism to be exploited (8, 9). Cell-free enzymatic cascade reactions can bypass many of the challenges that are typical for cell-based solutions. For instance, cell-free systems grant more freedom with respect to the reaction conditions, energy is not consumed to sustain the cell, product harvest is simpler, fewer by-products are produced, and cytotoxic compounds in any part of the cascade will not be an obstacle (6). The commercial feasibility of these systems is mainly governed by the cost to produce the enzymes. Hence, there is great interest in the ability to reuse complete enzyme cascades through enzyme immobilization in cell-free systems. This would increase the cost-effectiveness and could make cell-free systems industrially competitive.

Examples of demonstrated cascades include a combination of glucose oxidase (GOx)^2^ and horseradish peroxidase (HRP), which were bound onto DNA (10). It is worth noting that the combined enzymes show a spatial distance-dependent activity. To increase the enzymatic efficiency of the cascade, compartmentalization of lipase B, GOx, and HRP in polymersomes is another promising strategy (11). This applies also to GOx and HRP trapped in hydrogels (12). Another more advanced reaction cascade combines ribokinase, phosphoribosyl pyrophosphate synthetase, engineered hypoxanthine phosphoribosyl transferase, adenylate kinase, and pyruvate kinase to produce nucleotide analogues (13). In this setup, aggregated enzyme particles were created by precipitation with ammonium sulfate, followed by chemical crosslinking, which increased the stability compared with the soluble enzymes.

**Figure 1.**
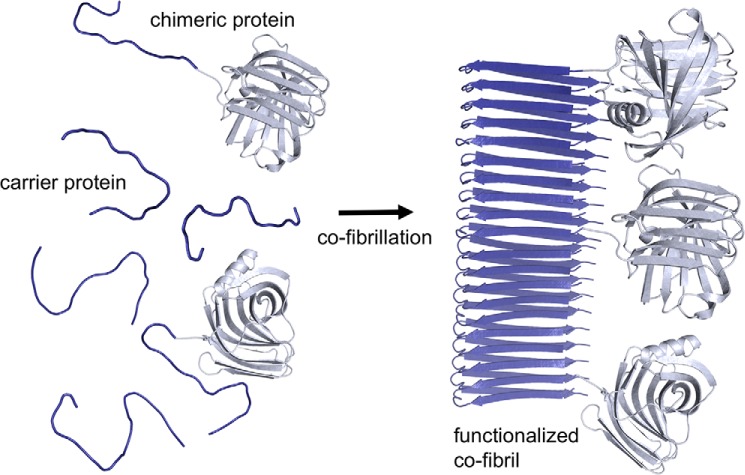
**Schematic outline of fibrillation to immobilize enzymes on protein nanofibrils.** A genetic fusion variant of the enzyme (*gray*) and an amyloidogenic peptide (*blue*), the chimeric protein is fibrillated together with carrier proteins, which is the amyloidogenic peptide alone. Co-fibrillation occurs spontaneously but can be accelerated by adding seeds at physiological pH. The final product is a fibril which has a diameter between 10 and 20 nm, and a length of more than 1 μm. The enzyme (xylanase A from *Bacillus subtilis*, PDB ID: 2Z79) is covalently displayed on the surface of the PNF. By adding carrier proteins during the fibrillation, enough space between the functional domains is introduced to eliminate steric restrictions.

Today, enzyme immobilization can be attained through a versatile toolbox, which includes chemical, photochemical, or adsorptive methods that tether the enzyme to appropriate materials (14, 15). Our approach to assemble an enzymatic cascade relies on covalent enzyme immobilization without using chemistry. To achieve this, we produced chimeric proteins, which are genetic fusions of enzymes and amyloidogenic peptides. The amyloidogenic peptide Sup35(1–61) (Sup35) is a truncated yeast transcription termination factor from *Saccharomyces cerevisiae,* which aggregates into highly ordered β-sheet secondary structures under physiological conditions (16, 17). Accordingly, the chimeric proteins self-assemble into protein nanofibrils (PNF), also known as amyloid, and display the enzymes homogenously on the surface (Fig. 1). The material displays a high surface-to-volume ratio, which ensures a high functional density.

The method of enzyme immobilization through protein display on PNFs is an established procedure and has been implemented with various enzymes in proof-of-concept studies (18–23). However, in most cases, the functionality is demonstrated on single-enzyme systems only. An exception is recently developed bacterial amyloid, which was used to assemble a two-enzyme cascade (24). Furthermore, the type of characterizations reported for enzyme functionalized amyloid have often a qualitative nature. This means that kinetic data for the optimization of the system with respect to the relative enzyme amount, pH, or temperature to maximize the product yield are missing. Nevertheless, kinetic characterization of the cascade is of great importance if the materials are developed for industrial processes.

The objective of this work was to fill this gap and develop a biocatalytic cascade, based upon PNF functionalized with enzymatic activities. Our concept relies on the processing of xylan from beechwood in three discrete enzymatic steps. Xylan is a highly abundant polysaccharide and constitutes up to 35% of plant cell walls (25). The wide availability of xylan renders it an appropriate sustainable resource, which is one of the key ingredients that accelerate the interest to utilize this biomass (26). Xylan is easily hydrolyzed to xylose with hydrochloric acid and heat, but biomass conversion using biocatalysts is acid-free and environmentally friendly. Thus, the biochemical method has a high potential to compete with the classical method, also in economic terms (27).

The enzymatic depolymerization of polysaccharides, for example, hemicellulose to monomeric sugars, requires a combined effort of xylanolytic enzymes (endoxylanase and β-xylosidase) (28). Hemicellulose is a branched heteropolymer consisting of pentose (xylose and arabinose) and hexose (mannose, glucose, and galactose) sugars, whereas xylose is the major component and constitutes the main chain (29). Because of the product inhibition caused by the xylooligosaccharides (xylobiose to xylopenatose (XOS)) produced by xylanase, β-xylosidases are usually considered the bottleneck in hemicellulose biodegradation (30).

## Results and discussion

### Fibrillation and characterization of the functionalized Sup35 nanofibrils

**Figure 2.**
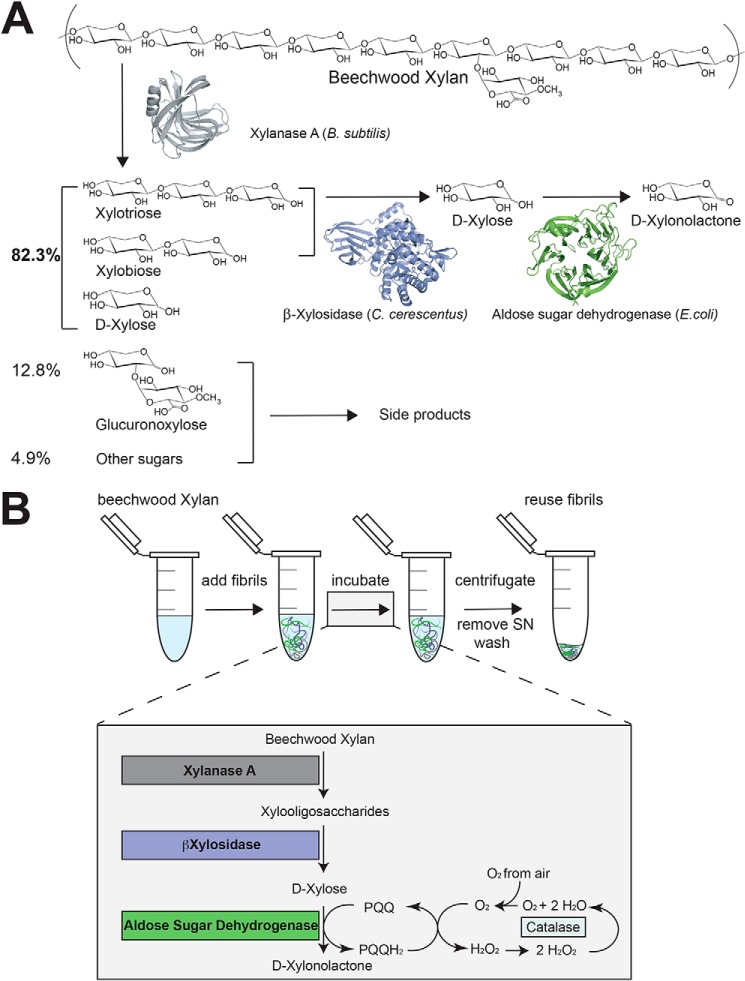
**Illustration of the reaction schemes of the enzyme cascade in this study.** *A*, xylanase A from *Bacillus subtilis* (PDB ID: 2Z79) releases xylose oligomers from xylan. Then, a β-xylosidase II from *Caulobacter crescentus* (PDB ID: 4EKJ) hydrolyzes the xylose oligomers into monomeric xylose. In the last step of the cascade, the aldose sugar dehydrogenase from *E. coli* (PDB ID: 2G8S) oxidizes xylose to xylonolactone. *B*, A Sup35 nanofibril suspension displays the enzymes and is incubated together with beechwood xylan. The oxidation of xylose requires the co-factor PQQ, which is reduced to PQQH_2_. PQQ is restored through oxidation by oxygen, which yields hydrogen peroxide. Once the entire reaction is complete the fibrils can be removed through centrifugation, the product can be harvested, and the fibrils can be reused.

To assemble the PNF-based cascade reaction, we chose to functionalize the PNF with enzymes that are monomeric and stable and that were previously produced in *Escherichia coli*. The enzymes xylanase A (XynA) from *Bacillus subtilis* (31), β-xylosidase II (βXyl) from *Caulobacter crescentus* (32), and a pyrroloquinoline quinone (PQQ)–dependent aldose sugar dehydrogenase (ASD) from *E. coli* (33) fulfil these requirements and were employed to create three Sup35–enzyme chimeras (*C*XynA, *C*βXyl, and *C*ASD, respectively) This combination hydrolyzes beechwood xylan into xylose monomers, which are subsequently oxidized into xylonolactone (Fig. 2). Xylonolactone is readily converted to xylonic acid by chemical or enzymatic means. Xylonic acid is a strategically important chemical for a bio-based economy and could, for instance, be used to synthesize co-polyamides (7, 34–36). To maximize the product yield, this three-step biocatalytic reaction was characterized with respect to the optimal pH, temperature stability, and relative enzyme concentration.

**Figure 3.**
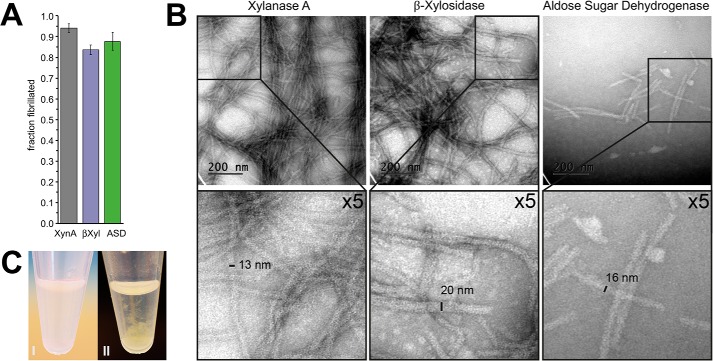
**Completeness of the fibril formation and TEM images of the enzymatic functionalized Sup35(1–61) fibrils.** *A*, the fraction of carrier and chimeric proteins that formed fibrils after adding seeds and a 24-h incubation. The values represent an average of five independent fibrillation reactions, and the *error bars* indicate the S.D. *B*, fibrils functionalized with XynA (*C*XynA^fib^) have a width of 13 nm. βXyl and ASD functionalized fibrils (*C*βXyl^fib^, *C*ASD^fib^) have a width of 20 and 16 nm, respectively. *C*, *C*ASD^fib^ fibrils before (I) and after (II) 5-min incubation at 37 °C.

Our method to functionalize Sup35 fibrils involves co-fibrillation of an amyloidogenic peptide (carrier protein) together with the chimeric construct by adding sonicated seeds. To fibrillate at least 85% after a 24-h incubation, we empirically optimized the fibrillation conditions by varying the relative ratio of seed, chimeric, and carrier protein (Fig. 3*A*). After their formation, the fibrils were imaged with transmission EM (TEM). The width of the fibrils was on average 13 nm (Sup35-xylanase A/Sup35 fibrils (*C*XynA^fib^)), 20 nm (Sup35–β-xylosidase II/Sup35 fibrils (*C*βXyl^fib^)), and 16 nm (Sup35-aldose sugar dehydrogenase/Sup35 fibrils (*C*ASD^fib^)) (Fig. 3*B*, the width of the Sup35 fibril core is 5 nm) (37). The difference in width corresponds well to the molecular mass of the protein chimeras, 29.8 kDa, 64.5 kDa, and 48.3 kDa for *C*XynA^fib^, *C*βXyl^fib^, and *C*ASD^fib^, respectively. It is also interesting to note that *C*XynA^fib^ and *C*βXyl^fib^ fibrils are longer than 1 μm, whereas *C*ASD^fib^ are much shorter and have a length between 90 and 400 nm. Because fibrils with a higher tensile strength grow longer (38), we speculate that *C*ASD^fib^ are shorter, because integrating *C*ASD into Sup35 fibrils may lead to a different folding pattern of the core compared with the other protein chimera.

Frequently, a reduced catalytic efficiency accompanies the display of enzymes on PNF (39). The origin of this effect is probably because of steric restrictions when only chimeric proteins are used for fibril assembly. Therefore, we assembled the fibrils with a reduced enzyme packing density (Fig. 1). In an earlier study, we had demonstrated that an apparent reduction of the catalytic efficiency of these fibrils in suspension can be attributed to mass transport limitations only (23). The mass transport limitations can be counteracted if the fibrils are trapped on a filter and the substrate is delivered by flow, not diffusion. Regarding *C*XynA^fib^ and *C*βXyl^fib^, *k*_cat_ and *K_m_* were comparable to the soluble enzyme (Tables 1 and 2). It was concluded that neither mass transport limitation nor steric restrictions significantly affected the enzyme. The same was true for *C*ASD^fib^, the catalytic efficiency *k*_cat_/*K_m_* of *C*ASD and *C*ASD^fib^ was almost identical. However, the fibrils did not show a particularly high tolerance toward increased temperature. At room temperature (RT), *C*ASD^fib^ were finely distributed, but immediately and irreversibly clumped together if they were incubated at 37 °C or if they were subjected to even mild agitation (100 rpm) (Fig. 3*C*).

**Table 1:** **Catalytic constants of soluble Sup35 fusion proteins** The error indicated is the S.D. of three experimental replicates.

Enzyme fusions	*k*_cat_	*K_m_*	*k*_cat_/*K_m_*
	*min*^−*1*^	*mm*	*mm*^−*1*^ *min*^−*1*^
*C*XynA*^a^*	1840 ± 35	0.3 ± <0.1	6053 ± 259
*C*βXyl*^b^*	1266 ± 55	7.4 ± 0.7	172 ± 10
*C*ASD*^c^*	285 ± 12	382 ± 45	0.75 ± 0.06

*^a^*37 °C, pH 6, substrate: XylX6.

*^b^*37 °C, pH 6, substrate: pNP-X.

*^c^*room temperature, pH 8.5, substrate: xylose.

**Table 2:** **Catalytic constants of enzymes displayed on Sup35 nanofibrils** The error indicated is the S.D. of three experimental replicates.

Enzyme fusions	*k*_cat_	*K_m_*	*k*_cat_/*K_m_*
	*min*^−*1*^	*mm*	*mm*^−*1*^ *min*^−*1*^
*C*XynA^fib^*^a^*	1147 ± 72	1.4 ± 0.2	851 ± 52
*C*βXyl^fib^*^b^*	1146 ± 81	2.2 ± 0.5	535 ± 95
*C*ASD^fib^*^c^*	106 ± 2	177 ± 11	0.60 ± 0.03

*^a^*37 °C, pH 6, substrate: XylX6

*^b^*37 °C, pH 6, substrate: pNP-X.

*^c^*room temperature, pH 8.5, substrate: xylose.

### Production of xylose by a CXynA/CβXyl enzyme mixture

**Figure 4.**
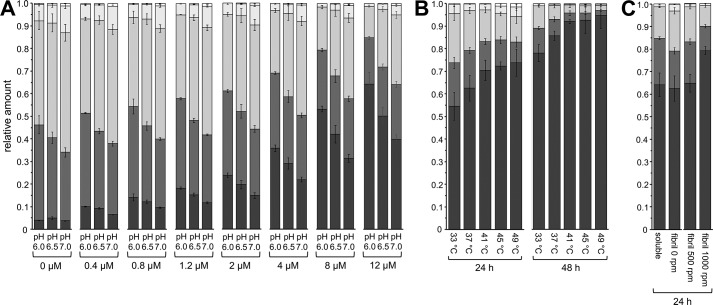
**Relative amounts (w/w) of the XOS after incubation of beechwood xylan with a *CXynA*/*C*βXyl enzyme mixture, determined by HPAEC.** *A–C*, the XOS, starting from xylose to xylopentaose were colored with a gradient from *dark gray* to *white*. The absolute amounts with S.D. of three independent experiments can be found in Tables S2–S4. *A*, soluble *C*XynA (0.4 μm) was mixed with *C*βXyl (0–12 μm) and incubated at pH 6.0, 6.5, and 7.0. The figure represents the XOS distribution after 24 h at 37 °C for each condition. *B*, a temperature gradient from 33–49 °C with *C*XynA^fib^/*C*βXyl^fib^ fibrils (0.4/12 μm enzyme concentration) after 24 and 48 h (see also Fig. S6). *C*, comparison of soluble enzymes against fibril with the same enzyme concentration *C*XynA/*C*βXyl (0.4/12 μm) after 24 h at 37 °C. The fibrils were also agitated at 500 and 1000 rpm. As a control, the same reaction was also performed using hybrid fibrils (*CXynA* and *C*βXyl were fibrillated together) instead of mixed fibrils (Fig. S5). The total substrate turnover yield for *A–C* is reported in Fig S4.

Prior to combining *C*XynA, *C*βXyl, and *C*ASD we wanted to achieve a better understanding of the interplay of *C*XynA and *C*βXyl that liberates xylose from xylan. Previous reports have found that pH 6.0 is the catalytic optimum for βXyl (32) and pH 7.0 for XynA (31, 40). To evaluate the efficacy of a combined pH optimum for *C*βXyl and *C*XynA we studied the time-dependent accumulation of xylose and XOS using pH values 6.0, 6.5, and 7.0. In addition, because we expected that the β-xylosidase would be limiting, the concentration of *C*βXyl was incrementally increased to reach a 30-fold molar excess over *C*XynA (Fig. 4*A* and Figs. S1–S3).

From these experiments, we could indeed conclude that the concentration of xylanase was not rate-limiting in the *C*XynA/*C*βXyl enzyme mixture with regard to xylose production. At a constant molar concentration of 0.4 μm *C*XynA in combination with 0–12 μm *C*βXyl, the xylose yield (24 h at 37 °C) steadily increased if more *C*βXyl was added to the reaction (Fig. S3**)**. This dependence was not linear, which implied that adding more than 12 μm would not accelerate the reaction substantially. For instance, in the absence of *C*βXyl, 4% of the total amount of the XOS is xylose, if the hydrolysis was carried out for 24 h at pH 6. A *C*βXyl concentration of 1.2 μm elevated the xylose fraction to 18%. However, 10-fold more *C*βXyl increased the xylose content only 3.5-fold to 64% (Fig. 4*A*).

The same conditions, but a higher pH (6.5, 7.0) resulted in reduced xylose production, although the overall XOS formation was not changed significantly. Consequently, the ratios of the formed XOS were skewed toward the longer XOS **(**Fig. 4*A***),** which is indicative of a loss of β-xylosidase activity only.

Next, we investigated how the enzymes would perform using optimal condition with regard to xylose production as determined by the previous assay (pH 6, 24 h, 37 °C, 0.4 μm *C*XynA, 12 μm *C*βXyl), but with the enzymes displayed on the fibrils. A comparison of the soluble enzyme with the fibril-catalyzed reaction revealed that the relative amounts of xylose and XOS were identical (Fig. 4*C*). In addition, the total product concentration of 3.1 mg/ml (Table S4) was identical between soluble and fibril-associated enzymes, which indicated that the enzyme activity was not significantly affected by immobilization on PNF.

**Figure 5.**
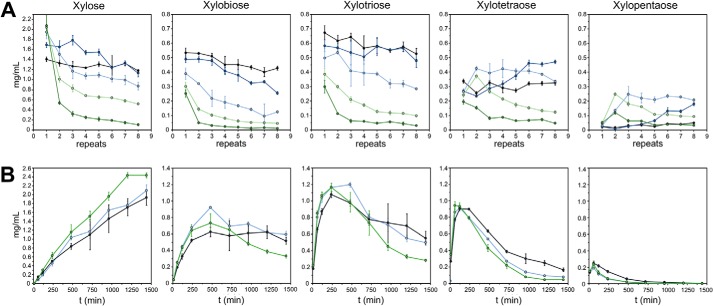
**The yield of XOS as a function of reaction repeats or time.** (A & B) The raw data with S.D. of three independent experiments can be found in Tables S5 and S6. *A*, the long-term stability of the *C*XynA^fib^/*C*βXyl^fib^ (0.4/12 μm) fibril mixture was studied using a temperature gradient from 33–49 °C, in 4 °C increments (*black* 33 °C, *dark blue* 37 °C, *light blue* 41 °C, *light green* 45 °C, *dark green* 49 °C). After each 24-h incubation, the fibrils were pelleted and washed before new substrate was added. *B*, to detect possible mass transport limitations of the *C*XynA^fib^/*C*βXyl^fib^ (0.4/12 μm) enzyme mixture, the accumulation of the XOS was studied over time, and by increasing the agitation of the sample vial (0 rpm *black*, 500 rpm *dark blue*, 1000 rpm *light blue*).

Another crucial characteristic of the fibrils is the temperature dependence of the *C*XynA^fib^/*C*βXyl^fib^ reaction, both in terms of xylose yield and long-term stability. If the fibrils are used only once, the relative xylose to XOS concentration is maximized at 49 °C (74% after 24 h and 95% after 48 h) (Fig. 4*B* and Fig. S7). Repeated recycling of the *C*XynA^fib^/*C*βXyl^fib^ fibril suspension resulted in a substantial decrease in the enzymatic activity over time at temperatures above 37 °C. Recycling the fibrils at 33 °C resulted in minimal losses of relative activity after eight consecutive 24-h reactions (Fig. 5*A* and Fig. S7), compared with the second round at 49 °C, which only yielded 25% of the xylose produced during the first round. Because the effect on xylose yield was greater than the effect on XOS accumulation, this highlighted the sensitivity of the catalysis to perturbations in the β-xylosidase activity. If the βXyl enzyme is not engineered or replaced, temperatures above 33 °C will only confer small benefits to the reaction yield if the fibrils are to be reused. However, if the fibrils are intended for single use, a temperature of 45 °C would be preferred, as it yielded the highest amount of xylose after 24 h (Fig. S3).

To elucidate possible mass transport limitations of the *C*XynA^fib^/*C*βXyl^fib^ enzyme mixture, product formation was analyzed over time with increasing agitation speed (Fig. 5*B*). As observed earlier, the total xylose yield was identical between soluble and fibril-associated *C*XynA^fib^/*C*βXyl^fib^ without agitation. However, the fraction of the XOS hydrolyzed to xylose is increased at 500 rpm. This increase was even more significant at 1000 rpm and corresponds to a 26% higher xylose concentration after 24 h at 37 °C (Figs. 4*C* and 5*B*). This mass transfer effect was probably caused by the heterogeneity of the beechwood substrate, because soluble enzyme and functionalized fibrils produced equal amounts of xylose at nonagitated conditions.

Natural and artificial alternatives to the reaction described here are supramolecular complexes referred to as xylanosomes (41–43). However, these complexes are poorly characterized in terms of their activity, which means that a proper comparison to the *C*XynA^fib^/*C*βXyl^fib^ enzyme system is not possible at this point.

### Factors contributing to the oxidation of xylose by CASD

**Figure 6.**
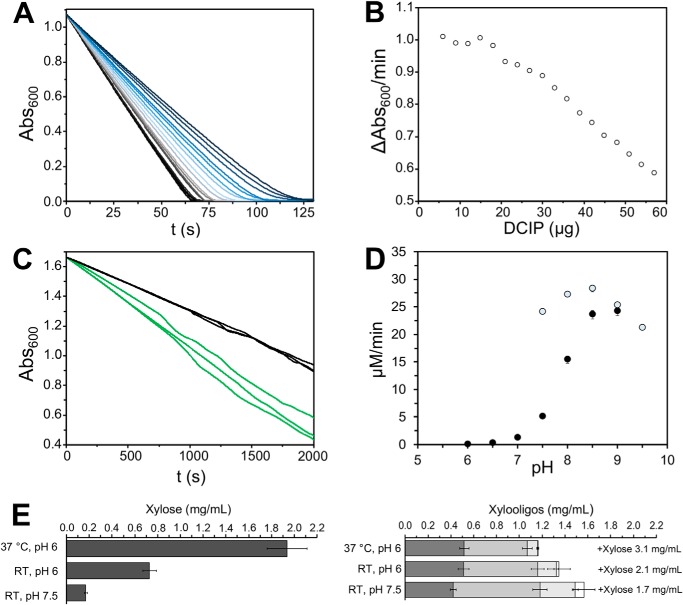
**Conditions that influence the enzymatic activity of *C*ASD.** *A* and *B*, incremental addition of DCIP to a 1-ml cuvette containing 100 mm xylose and 8 μm soluble *C*ASD. *A*, the lines were colored using a gradient from *black* to *dark blue*, indicating the increasing DCIP amount in the cuvette between 6 μg (*black*) and 60 μg (*dark blue*). *B*, the activity decreases significantly when more than 15 μg DCIP is present in the cuvette. *C*, comparison of the rate of xylose oxidation without (*black*) and with catalase (*green*). *D*, the initial velocity of xylose oxidation with ASD at RT and 100 mm xylose, as a function of the pH. *Black circles* refer to measurements using 100 mm sodium phosphate buffer, at pH 8.5 and pH 9.0 in the presence of 100 mm Tris. *Empty circles* were measured using 100 mm Tris buffer. The raw data with S.D. of three independent experiments can be found in Table S7. *E*, preparatory experiment to combine *C*XynA^fib^/*C*βXyl^fib^ and *C*ASD^fib^ fibrils. Xylose and XOS yield after 24 h using *C*XynA^fib^/*C*βXyl^fib^ fibrils. A change from 37 °C to RT at pH 6.0 reduces the yield of xylose by a factor of 2.6. Changing the pH from 6.0 to 7.5 reduces the xylose yield additionally by a factor of 4.2. The total yield of xylobiose, xylotriose, xylotetraose, and xylopentaose (*dark gray* to *white*) is only reduced by a factor of 2 between 37 °C, pH 6 and RT, pH 7.5. The raw data with S.D. of three independent experiments can be found in Table S8.

To attain a complete conversion of xylose to xylonolactone, we investigated the factors that influence the efficiency of *C*ASD. Standard ASD activity assays employ 2,6-dichlorophenolindophenol (DCIP), which acts as a hydrogen acceptor of reduced pyrroloquinoline quinone (PQQH_2_). Reduced DCIP switches color from dark blue to colorless, which makes it a suitable indicator for observation of xylonolactone accumulation in real time. Because the amount of DCIP in an activity assay is normally very small compared with xylose released by *C*XynA^fib^/*C*βXyl^fib^ fibrils, we first investigated how the reaction is affected if more DCIP is added (Fig. 6, *A* and *B*). Our results indicated that the reaction is inhibited significantly above 15 μg/ml DCIP. We, therefore, decided not to include DCIP in our final setup.

We hypothesized that the reaction will cease when the accumulation of H_2_O_2_ by oxidation of PQQ begins to favor the reverse reaction. In an attempt to eliminate the chance of a reverse reaction, we tested how the addition of catalase, which converts hydrogen peroxide to H_2_O and O_2_, would affect the rate of catalysis (Fig. 6*C*). We used a very low *C*ASD concentration and observed an increased rate of xylose oxidation by 60% upon catalase addition. Catalase was therefore included in all reactions that involved *C*ASD.

To identify a suitable pH value for combining *C*XynA/*C*βXyl/*C*ASD in a single reaction, we first determined the relative activity of *C*ASD at 100 μm xylose (below *K_m_*) as a function of the pH (Fig. 6*D* and Table S1). In our enzyme cascade, *C*ASD was already the enzyme with the lowest catalytic efficiency, but decreasing the pH from 9.0 to 6.0 reduced the rate of catalysis of the soluble enzyme by a factor of 300. Therefore, a compromise with respect to pH is the only option for an enzyme mixture that contains *C*XynA/*C*βXyl/*C*ASD.

### The three-enzyme reaction cascade

Considering these results, combining all three enzymatically functionalized fibrils in one unified reaction proved to be challenging. To proceed, there were two strategies to obtain xylonolactone from xylan: First, a fibril suspension that includes all enzymes with reaction conditions known to be suboptimal, but would require less sample handling. Second, the reaction could be executed using a modular reaction cascade. During the first stage, xylan is hydrolyzed to xylose with *C*XynA^fib^/*C*βXyl^fib^ fibrils. The fibrils are then removed, the product is recovered and the pH is finally adjusted before *C*ASD^fib^ fibrils are added to oxidize xylose to xylonolactone. The advantage of this setup is that the conditions can be optimized for each enzymatic step, the complete turnover is much faster, and the amount of enzyme is reduced.

**Figure 7.**
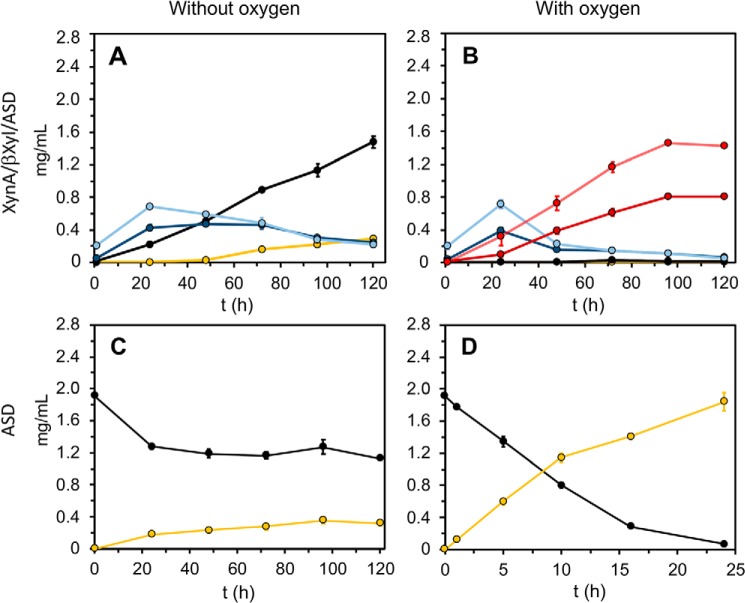
**Production of xylonolactone at RT (*black*: xylose, *dark blue*: xylobiose, *light blue*: xylotriose, *yellow*: xylonolactone).** *A–D*, all values are averages of three replicate experiments. The raw data with S.D. can be found in Tables S9–S12. *A* and *B*, a fibril mix containing 0.4 μm *C*XynA^fib^, 12 μm *C*βXyl^fib^, and 120 μm *C*ASD^fib^. Substrate: 1% beechwood xylan, pH 7.5. *B*, xylobionolactone (*dark red*) and xylotrionolactone (*light red*). The method for quantification is described in the supporting information (Fig. S9). *C* and *D*, the sample contains only ASD functionalized fibrils (40 μm). The substrate was prepared by hydrolyzing beechwood xylan using the 0.4 μm *C*XynA^fib^/12 μm *C*βXyl^fib^ enzymatic fibril mixture at 37 °C and pH 6.0. Before *C*ASD^fib^ fibrils were added, *C*XynA^fib^/*C*βXyl^fib^ fibrils were removed and the pH was adjusted to 9.0. This reaction was also repeated using lower concentrations of *C*ASD^fib^. As expected, the rate of xylose oxidation is linear dependent upon ASD concentration (Fig. S10). *Without oxygen* refers to incubation in a sealed Eppendorf vial. *With oxygen* means that three holes were introduced into the Eppendorf vial to ensure oxygenation of the sample.

Regarding the first strategy, a cascade containing a combination of all three enzymes at pH 6 is not a viable option, because 300-fold more *C*ASD^fib^ would be required to compensate the loss of catalytic activity compared with pH 9.0. Therefore, we investigated if a compromise at pH 7.5 would be more appropriate for the triple enzyme cascade. In addition to determining *C*XynA^fib^/*C*βXyl^fib^ fibril performance at pH 7.5, we also had to consider that a combined reaction has to be executed at RT, to ensure the integrity of the *C*ASD^fib^ fibrils. To obtain comparable results with respect to data from the xylan hydrolysis at 37 °C and pH 6.0, we determined the xylose yield using the *C*XynA^fib^/*C*βXyl^fib^ suspension (0.4/12 μm), but at RT and at pH 6.0 as well as pH 7.5 (Fig. 6*E* and Fig. S8). This experiment demonstrated that the xylose yield is reduced by a factor of 11 following a 24-h incubation. The data also suggested that pH 7.5 and RT affect *C*βXyl^fib^ more than *C*XynA^fib^ because the total amount of XOS released is only reduced 2-fold. To compensate for the reduced production efficiency of xylose, we decided to increase the incubation time (Fig. 7, *A* and *B*).

The second strategy for producing xylonolactone benefited from the fact that no common denominator for the conditions has to be identified. The product from *C*XynA^fib^/*C*βXyl^fib^ mixture at 37 °C served directly as the substrate for ASD after the pH has been adjusted to 9.0 (Fig. 7, *C* and *D*).

In both instances, we immediately observed that the oxidation of xylose by *C*ASD^fib^ fibrils is very inefficient in a sealed Eppendorf vial (Fig. 7, *left column*). Without oxygen, the yield of xylonolactone using setup 2 is less than 0.4 mg/ml (16% of the total xylose amount) after incubation with 40 μm *C*ASD^fib^ for 5 days. In fact, xylose oxidation essentially stopped after 48 h. Likewise, the xylonolactone production in setup 1 does not start before 48 h have passed, and after 5 days only 0.3 mg/ml have accumulated. Because oxygen is required to recycle the reduced cofactor PQQH_2_, we expected that limited oxygenation of the sample could cause the slow progression and premature end of the catalysis. To allow fresh air to access the sample constantly, three holes were introduced to the reaction container, two on the side and one on the top (Fig. 7, *right column*).

Then, the same amount of ASD functionalized fibrils (setup 2) oxidize 97% of the xylose to xylonolactone during 24 h of incubation. In addition, we have also confirmed that the fibrils can be reused three times before the enzymes gradually deteriorate (Fig. S11).

The success of producing xylonolactone in the oxygenated triple enzyme setup could be improved. ASD is known to have a broad substrate specificity and oxidizes monosaccharides, disaccharides, and trisaccharides (33). Therefore, we expected that xylobiose and xylotetraose would quickly be oxidized in this setup (Fig. 7*B*). However, because xylonolactone does not accumulate, we speculated that *C*βXyl is not able to hydrolyze xylobionolactone and xylotrionolactone. Thus, setup 1 requires further optimization and a substantial increase of *C*XynA^fib^/*C*βXyl^fib^ fibrils to shift the equilibrium toward the production of xylonolactone.

### Biomass processing and biotechnological applications

The industrial synthesis of high-value chemicals, such as pharmaceutical compounds, is in the majority of cases based on nonreusable catalysts as the costs of these catalysts is just a fraction of the final selling price of the product (45). However, low-value chemicals that are needed in large quantities, such as second-generation biofuels, sweeteners, and alcohols, would benefit from biocatalytic synthesis pathways that employ reusable enzymes to make commercially competitive products.

Xylonic acid is one such compound. A biocatalytic pathway based on the setup design described in this work could yield an acid and alkaline-free, environmentally friendly alternative to the chemical synthesis of xylonic acid. This study can also contribute to increased productivity of xylan biomass hydrolysis for the purpose of second-generation biofuel production. Sustainable biofuel production is based upon the release of monomeric sugars from long-chained carbohydrates. This process requires xylanases and xylosidases (46–48), which contribute to the hydrolysis of cellulose because cellulases are otherwise inhibited by the presence of xylan (49). The enzymes currently used in biofuel production account for ∼40% of the process cost (50). Therefore, reusable enzymes could significantly contribute to reducing the current cost of second-generation biofuels.

To make this possible, we have recently described a method to produce ready-to-use functionalized nanofibrils with *Komagataella pastoris*, which can be separated from the cells using only centrifugation and saline (51). This concept could also be applied to express the enzymes needed for industrial biocatalytic pathways and attain their immobilization on PNF simultaneously. Generally, a major drawback of enzyme immobilization for industrial-size applications is the additional cost of the immobilization matrix and the crosslinking chemicals (45). If enzymatic ready-to-use nanofibrils can be produced in significant quantities with our method, these costs could become negligible. Considering all these factors, functionalized amyloid fibrils are a promising concept for the design of biocatalytic cascades.

## Conclusion

We believe that this work is evidence for the feasibility of functionalized Sup35(1–61) fibrils as a biocatalytic cascade concept. Using the cell-free setup presented in this paper, we were able to adjust the relative ratios of the enzymes to maximize our product yield, which is not easily accomplished in engineered whole-cell biocatalysts. Furthermore, the enzymatically endowed fibrils can be reused, which has the potential to alleviate the economic impact of preparing low-value chemicals.

We have also demonstrated that the enzymes do not lose activity when displayed on nanofibrils compared with their native state and show that the viability of the Sup35 fibrils as enzyme scaffolds depends on the stability of the immobilized enzyme. Therefore, an efficient catalysis still requires the use of engineered enzymes to increase the robustness of our catalytic cascade.

Changes in the assay conditions showed that small perturbations affect the substrate yield of the *C*XynA^fib^/*C*βXyl^fib^ enzyme mixture. Therefore, the data of this work are a suitable template for other improved designs of this xylan processing enzyme cascade. Ultimately, an industrial-sized application will be governed by economic factors, which determine a reasonable balance between the enzyme amount and the time necessary to reach the desired product concentration.

## Experimental procedures

### Cloning

The coding sequences for XynA (GenBank^TM^ accession no. CAA84276.1, aa 29–213), βXyl (PDB ID: 4EKJ, aa 1–499), and ASD (GenBank^TM^ accession no. WP_024244531.1, aa 21–370) were codon-optimized for expression in *E. coli*, and synthesized by Integrated DNA Technologies (Coralville, IA). The lyophilized DNA was re-suspended in water and cleaved with the restriction enzymes XhoI/BamHI (XynA, ASD) or XbaI/BamHI (βXyl). The vector construct pET-28b(+) (Novagen) which contained an N-terminal His_6_ tag, a thrombin cleavage site, Sup35(1–61) (37), and a GGGGSG linker was digested with the same enzymes. The vector backbone and the new inserts were ligated with T4 DNA ligase (Thermo Fisher) according to the protocol from the manufacturer. The ligation product was used to transform chemically competent *E. coli* Top10 (Thermo Fisher). Colonies on selective LB-agar plates (50 μg/ml kanamycin) were cultured overnight in LB-medium at 25 °C, 180 rpm (50 μg/ml kanamycin). Then, the GeneJET Plasmid Miniprep Kit (Thermo Fisher) was used to purify the new plasmids. The plasmids were sequenced by Macrogen Europe (Amsterdam, Netherlands). Finally, the plasmids pET-28b(+) with the genes for *C*XynA, *C*βXyl, and *C*ASD were used to transform *E. coli* BL21 Star (DE3) chemically competent cells (Thermo Fisher) according to the protocol from the supplier.

### Protein expression and purification

One colony of transformed *E. coli* BL21 Star (DE3) was used to inoculate 2 ml of terrific broth (TB)–medium (kanamycin 50 μg/ml). The pre-culture was grown at 37 °C and 180 rpm for 4 h. Subsequently, the pre-culture was used to inoculate 900 ml of TB-medium, followed by incubation at 37 °C, 160 rpm, until the *A*_600_ reached 1. Then, the culture was cooled on ice, before isopropyl 1-thio-β-D-galactopyranoside was added to a final concentration of 0.1 m. Protein expression was carried out at 180 rpm and 16 °C for 20 h. Next, the cells (*A*_600_ > 10) were harvested at 5000 × *g* for 20 min in a Sorvall LYNX 6000 (Thermo Scientific) centrifuge and an F9–6 × 1000 LEX rotor. The cell pellet was re-suspended by gentle agitation in 30 ml lysis buffer (20 mm NaP_i_ for *C*XynA and *C*βXyl; 25 mm HEPES for *C*ASD; 150 mm NaCl; pH 7.4). Cell lysis was executed in a cell disruptor (Constant Systems) at 1,350 bar. After cell disruption, the lysate was centrifuged at 38,000 × *g* in a Sorvall RC 6+ (Thermo Scientific) using an F21–8 × 50y rotor (4 °C for 30 min). Then, one tablet cOmplete protease inhibitor (Roche) and DNase I to a final concentration of 10 μg/ml were added to the lysate. The lysate was incubated on ice for 30 min and afterwards filtered through a Filtropur S 0.45 μm (Sarstedt). All subsequent purification steps were carried out on an Äkta Explorer liquid chromatographic system (GE Healthcare). A Ni^2+^-charged 5-ml HiTrap Chelating HP (GE Healthcare) column was equilibrated with Binding Buffer (BB) (20 mm NaP_i_, pH 7.4 for *C*XynA and *C*βXyl or 25 mm HEPES, pH 7.4 for *C*ASD) before the protein was loaded. Next, the protein-binding column was washed with BB (20 mm NaP_i_ for *C*XynA and *C*βXyl, 25 mm HEPES for *C*ASD) containing 7.5% Elution Buffer (BB with 400 mm imidazole). Subsequently, the *C*XynA, *C*βXyl or *C*ASD were eluted with 75% Elution Buffer. Finally, the eluted fractions were analyzed with SDS-PAGE. The fractions containing the protein of interest were pooled and desalted with a PD10 desalting column (GE Healthcare) into 100 mm NaP_i_, pH 6 (*C*XynA), 20 mm NaP_i_, pH 7.4 *C*βXyl), or 25 mm HEPES (*C*ASD). The purified proteins were aliquoted, flash frozen with liquid nitrogen, and stored at −80 °C. The carrier protein Sup35(1–61) was produced as reported previously (37).

### Fibrillation

Co-fibrillation to create doped nanofibrils was essentially performed as described previously in fibrillation buffer (30 mm Tris, 200 mm NaCl, pH 8) (23, 37). Briefly, seeds to start the fibrillation of carrier protein were created by 6-min sonication of a 1.5-ml Eppendorf tube using a Branson 2510 Ultrasonication bath. After sonication, the seeds were instantaneously transferred to a fresh, sterile Eppendorf tube containing soluble carrier and chimeric proteins. To create *C*XynA-doped Sup35 nanofibrils, 23 μg seed was added to 2 μg carrier protein and 61 μg chimeric protein. The fibril had a doping frequency of 1:0.74 with respect to the molar concentration of carrier over the chimeric protein. In the same manner, *C*βXyl-doped Sup35 nanofibrils were assembled by addition of 11 μg seed to 23 μg carrier protein and 81 μg chimeric protein. The doping frequency of the fibril was 1:0.33.

*C*ASD was incubated with a 10-fold molar excess of PQQ and 1 mm CaCl_2_ at ambient temperature. Then, *C*ASD and Sup35 were co-fibrillated by transferring 16 μg seed to 2 μg carrier protein and 82 μg chimeric protein. Here, the doping frequency of the fibril was 1:0.83. After addition of the seeds, the fibrils were allowed to assemble by overnight incubation at ambient temperature. The completeness of the fibrillation was assessed by pelleting the fibrils using centrifugation at 5000 × *g* for 10 min in a Heraeus Pico 17 bench-top centrifuge (Thermo Fisher). Subsequently, the absorbance of the supernatant was measured at 280 nm (*A*_280_) using a NanoDrop 1000 (Thermo Fisher) and compared with the *A*_280_ before fibrillation. In the case of *C*ASD^fib^, we used SDS-PAGE to estimate the completeness of the fibrillation by peak integration of the protein bands corresponding to the total protein content and the nonfibrillated fraction. Before using the fibrils for enzyme kinetic studies, they were pelleted and washed three times using water, or a buffer that matches the components of the enzyme assay.

### Enzyme kinetic assays using a spectrophotometer

The enzymatic activity of *C*XynA was assayed using the artificial 4,6-*O*-(3-ketobutylidene)-4-nitrophenyl-β-d-4^5^-glucosyl-xylopentaoside (XylX6) substrate, which is specific for xylanase hydrolysis (Megazyme) (52). Initial rates of XylX6 hydrolysis were determined using a UV-1700 PharmaSpec (Shimadzu) spectrophotometer. The reaction was initiated by *C*XynA (final concentration 7.5 nm) to 0.03–2 mm XylX6 (2-fold dilution series), in a 0.1 m NaP_i_ buffer (pH 6) containing β-xylosidase. After 10-min incubation at 37 °C, the reaction (total volume 100 μl) was stopped by adding 2% Tris, pH 10, 1500 μl. The substrate-specific extinction coefficient Δϵ = 18,100 m^−1^ cm^−1^ was used to convert *A*_400_ values to molar concentrations_._ The same setup was used to determine the catalytic constant of *CXynA*^fib^ fibrils, but with a 20 nm enzyme concentration.

The initial reaction velocity of pNP-X (Sigma) hydrolysis was assayed using *C*βXyl in a 0.1 m NaP_i_ buffer (pH 6). The enzyme concentration in the assay was 10 nm, both for soluble enzymes and immobilized on the fibril. The substrate concentration was varied between 0.5 and 20 mm in 1 mm increments. After incubation of 150 μl for 10 min at 37 °C in a clear F-bottom 96-well plate (Nunc), an equal volume of 0.5 m Na_2_CO_3_ was added to end the reaction. The *A*_400_ was measured using an Eon (BioTek) microplate reader. The substrate specific extinction coefficient for the reaction is Δϵ = 18,100 m^−1^ cm^−1^.

*C*ASD was incubated with a 10-fold molar excess of PQQ (Sigma) and 1 mm CaCl_2_. After incubation at RT for 1 h, the enzyme was desalted into 25 mm HEPES, pH 7.5, using a PD10 column. A typical assay was carried out in 50 mm KP_i_ (pH 6.5–7.5) and a 2-fold dilution series of xylose in the range of 0.31–4 M. In addition, the reaction mixture (1 ml) contained 5.4 μm PQQ (Sigma), 10 μg/ml catalase (Sigma), and 4.7 μm DCIP (Sigma). The linear reduction of DCIP to DCIPH_2_ was continuously monitored at 600 nm for at least 60 s (Δϵ = −21,000 m^−1^ cm^−1^). The concentration of the soluble enzyme in the assay was pH-dependent, but between 75 nm and 250 nm. The same was true for activity measurements using the *C*ASD^fib^ fibrils to obtain a final enzyme concentration of 150–300 nm. All enzyme kinetic experiments were done in triplicates. The data kinetic constants were determined by nonlinear regression using the mmfit and rffit programs, which are included in the SimFit software (54).

### Assays of the enzyme cascade reactions

The enzyme substrate, beechwood xylan (Megazyme), was wetted with 95% ethanol and then diluted to a final concentration of 2% beechwood xylan, 16% ethanol with a 0.2 m KP_i_ buffer, pH 6.0–7.5. The solution was heated to 120 °C for 10 min to solubilize xylan and subsequently cooled to RT before the pH was readjusted. Finally, the solubilized xylan was filtered through a Filtropur S 0.2 μm (Sarstedt) filter and 0.05% toluene was added to inhibit microbial growth. To assess the total content of xylose in the substrate solution, it was diluted 50 times in HCl (pH 0.5) and heated at 90 °C for 24 h (53).

To characterize the xylose production from beechwood xylan, soluble *C*XynA was diluted to 0.4 μm, together with *C*βXyl in the range of 0–12 μm. The final substrate concentration was 1% beechwood xylan, also containing 8% ethanol, in a 0.1 m KP_i_ buffer (pH 6.0–7.5). The 100 μl reaction cocktails were incubated in Multiply–μStrip Pro PCR tubes (Sarstedt) with a safety lock, at 37 °C in a Veriti 96-well Thermal Cycler (Applied Biosystems), with a heated lid (80 °C). Samples were removed after 10, 60, 120, 240, 480, 720, 960, 1200, and 1440 min, with an eight-channel multipipette and diluted 40 times into 0.1 m NaOH and stored at −20 °C.

The enzymatic xylan hydrolysis reaction was also characterized with respect to the influence of the temperature. Here, *C*XynA^fib^ and *C*βXyl^fib^ fibrils that yield an enzyme concentration of 0.4 μm and 12 μm, respectively, were incubated with xylan (1% xylan, 8% ethanol, 0.1 m KP_i_, pH 6) at 33–49 °C, in 4 °C increments. Otherwise, an identical experimental setup as described above was used to obtain comparable results. After 1440 min had passed, the fibrils were pelleted, and the supernatant was discarded. The fibrils were washed with 0.1 m KP_i_, pH 6. A new reaction was started by adding the beechwood xylan to a final concentration of 1%. In total, the reaction was repeated eight times to assess the thermal stability of the enzyme mix. Samples were only removed after a complete 1440-min cycle.

To study the effect of possible mass transport limitations, the *C*XynA^fib^ and *C*βXyl^fib^ fibrils were subjected to 500 and 1000 rpm in a 2-ml Eppendorf vial using a Thermo MixerC (Eppendorf). Analogous to the conditions used previously, the reaction was assessed using 1% xylan (8% ethanol, 0.1 m KP_i_, pH 6) at 37 °C, and a total volume of 100 μl. Likewise, the final enzyme concentration was 0.4 μm and 12 μm, for *C*XynA^fib^ and *C*βXyl^fib^, respectively.

To study the three-enzyme reaction cascade, with the aim to hydrolyze xylan (beechwood xylan 1%, pH 7.5) to xylose, and to oxidize xylose to xylonolactone, *C*XynA^fib^ (0.4 μm), *C*βXyl^fib^ (12 μm), and *C*ASD^fib^ (120 μm) fibrils were incubated in 2-ml Eppendorf tubes at RT. Samples were removed in 24-h increments. As a reference, *C*XynA^fib^ (0.4 μm) and *C*βXyl^fib^ (12 μm) fibrils were incubated with the substrate without *C*ASD^fib^, at pH 6.0 and at pH 7.5.

The *C*ASD substrate for the modular reaction cascade was produced by incubating 1% beechwood xylan, pH 6.0 with *C*XynA^fib^/*C*βXyl^fib^ (0.4/12 μm) fibrils at 37 °C for 24 h. After incubation, the fibrils were pelleted and the product was recovered. The fibrils were washed and reused. All product fractions were pooled and heated to 70 °C to remove water. Then, the pH was adjusted to 9.0 with 100 mm tris-HCl and water was added to dilute the sample to the original volume. *C*ASD^fib^ fibrils (40 μm enzyme) were incubated with the substrate at RT. To test the fibril reusability, after 24 h the *C*ASD^fib^ fibril suspension was centrifuged at 5000 × *g* and the supernatant was removed. Subsequently, the fibrils were washed with 100 mm Tris, pH 9, before a new substrate was added. All of the reactions described in this section were executed in triplicates.

### Analysis of reaction products of the enzyme cascade using HPAEC

The samples from the enzyme cascade assays were prepared by 10-fold dilution into 0.1 m NaOH. The carbohydrate content of the samples was quantified by high-performance anion exchange chromatography (HPAEC). The HPAEC analyses were performed using a CarboPac PA1 2 × 250 mm analytical column (Dionex Corp., Sunnyvale, CA) mounted in an ICS3000 system equipped with a pulsed amperometric detector (Dionex Corp., Sunnyvale, CA) and a 5-μl sample loop. A gradient elution program was applied after a full-loop injection in each analysis. The elution program included the following steps applying a 0.25 ml/min flow: First, a 15-min gradient from 0.1 m NaOH to 0.1 m NaOH and 0.15 m NaAc was used. This gradient was followed by a steeper 3-min gradient to 1 m NaAc. Then, the column was treated for an additional 10 s with 1 m NaAc before the column was re-equilibrated for 5 min using 0.1 m NaOH. To quantify the XOS, known amounts of xylose, xylobiose, xylotriose, xylotetraose, and xylopentaose (Megazyme), as well as xylonolactone (Sigma), were used as standards (a 2-fold dilution series in the range of 0.08–20 μg/ml).

### Transmission electron microscopy

After fibrillation, the fibrils were washed two times by centrifugation at 5000 × *g* and resuspension in fibrillation buffer. Then, the fibrils were deposited on carbon-coated grids (SPI Supplies) and negatively stained with 1% uranylacetate (44) before they were imaged by staff from the BioVis facility at the Rudbeck Laboratory (Uppsala University, Uppsala, Sweden). The width and length of the fibrils were estimated using Photoshop CC (Adobe).

## Author contributions

B. S., M. G., and M. S. conceptualization; B. S. and M. G. data curation; B. S. and M. G. formal analysis; B. S. and M. G. validation; B. S. and M. G. investigation; B. S. and M. G. visualization; B. S., M. G., and M. S. methodology; B. S. and M. G. writing-original draft; B. S., M. G., T. H., and M. S. writing-review and editing; T. H. and M. S. resources; T. H. and M. S. supervision; T. H. and M. S. project administration.

## Supplementary Material

Supporting Information

## Footnotes

2 The abbreviations used are:

GOx: glucose oxidase
aa: amino acid
ASD: aldose sugar dehydrogenase
BB: Binding Buffer
DCIP: 2,6-dichlorophenolindophenol
HPAEC: high-performance anion exchange chromatography
PNF: protein nanofibrils
PQQ: pyrroloquinoline quinone
PQQH_2_: reduced pyrroloquinoline quinone
RT: room temperature
Sup35: Sup35(1–61)
TEM: transmission EM
XOS: xylobiose to xylopenatose
βXyl: β-xylosidase II
XynA: xylanase A.
