# Supplementary material for: Coupled chemistry kinetics demonstrate the utility of functionalized Sup35 amyloid nanofibrils in biocatalytic cascades
Source: J Biol Chem. 2019 Aug 15;294(41):14966–77. doi: 10.1074/jbc.RA119.008455 (PMC6791322; doi:10.1074/jbc.RA119.008455)
Supplement: Supporting Information [file supp_294_41_14966__index.html]

Coupled chemistry kinetics demonstrate the utility of functionalized Sup35 amyloid nanofibrils in biocatalytic cascades — Kinetics of Functionalized Sup35 Amyloid Nanofibrils — Coupled chemistry kinetics demonstrate the utility of functionalized Sup35 amyloid nanofibrils in biocatalytic cascades — Kinetics of functionalized Sup35 amyloid nanofibrils — Supporting Information 

# Coupled chemistry kinetics demonstrate the utility of functionalized Sup35 amyloid nanofibrils in biocatalytic cascades

## Supporting Information

- Supporting Information - Table S1-S12 and Figure S1-S11
