# Supplementary material for: Coupled chemistry kinetics demonstrate the utility of functionalized Sup35 amyloid nanofibrils in biocatalytic cascades
Source: J Biol Chem. 2019 Aug 15;294(41):14966–77. doi: 10.1074/jbc.RA119.008455 (PMC6791322; doi:10.1074/jbc.RA119.008455)
Supplement: Supporting Information [file supp_RA119.008455_144331_3_supp_378687_pw82vl.pdf]

Supporting Information for

Coupled Chemistry Kinetics of Functionalized Sup35 Amyloid Nanofibrils

Benjamin Schmuck†, Mikael Gudmundsson†, Torleif Härd†, and Mats Sandgren†*

†Department of Molecular Sciences, Swedish University of Agricultural Sciences, Box 7015, 756 51 Uppsala, Sweden

*Correspondence to: mats.sandgren@slu.se

Table S1. Catalytic constants of soluble Sup35-ASD fusion proteins.

Sup35-ASD	k_{cat} (min^{-1})	K_M (M)	k_{cat} / K_M (M^{-1} min^{-1})
pH 7.5[a]	299 ± 23	1.6 ± 0.3	181 ± 18
pH 7.0 [a]	93 ± 5	1.5 ± 0.1	64 ± 5
pH 6.5[a]	30 ± 3	4.7 ± 0.7[b]	6 ± 1

All measurements were performed in potassium phosphate buffer. [a] RT, Substrate: xylose. The error indicated is the standard deviation of three experimental replicates. [b] Theoretical value, since the K_M is above the solubility of xylose. The catalytic constants at pH 6.0 were not determined, since the theoretical K_M already at pH 6.5 is above the limit of solubility of xylose.

Table S2. Raw data with standard deviations for figure **4A**

		0 µM (mg/ml)	0.4 µM (mg/ml)	0.8 µM (mg/ml)	1.2 µM (mg/ml)	2 µM (mg/ml)	4 µM (mg/ml)	8 µM (mg/ml)	12 µM (mg/ml)
pH 6	X1	0.12 ± 0.01	0.32 ± 0.01	0.47 ± 0.06	0.57 ± 0.02	0.78 ± 0.04	1.19 ± 0.05	1.76 ± 0.05	2 ± 0.17
	X2	1.21 ± 0.12	1.3 ± 0.01	1.35 ± 0.11	1.24 ± 0.02	1.22 ± 0.03	1.1 ± 0.02	0.87 ± 0.03	0.65 ± 0.03
	X3	1.31 ± 0.12	1.31 ± 0.03	1.32 ± 0.1	1.16 ± 0.01	1.11 ± 0.03	0.91 ± 0.03	0.63 ± 0.03	0.44 ± 0.02
	X4	0.21 ± 0.02	0.2 ± 0.02	0.2 ± 0.01	0.16 ± 0.01	0.17 ± 0.03	0.11 ± 0.02	0.06 ± 0.01	0.04 ± 0.01
	X5	0.02 ± 0.01	0.02 ± 0.01	0.02 ± 0.01	0 ± 0	0 ± 0	0 ± 0	0 ± 0	0 ± 0
pH 6.5	X1	0.17 ± 0.02	0.34 ± 0.02	0.46 ± 0.03	0.56 ± 0.03	0.75 ± 0.07	1.06 ± 0.09	1.6 ± 0.15	1.89 ± 0.15
	X2	1.14 ± 0.09	1.25 ± 0.05	1.25 ± 0.07	1.22 ± 0.04	1.22 ± 0.12	1.08 ± 0.1	0.98 ± 0.11	0.82 ± 0.06
	X3	1.62 ± 0.13	1.81 ± 0.07	1.77 ± 0.09	1.68 ± 0.05	1.6 ± 0.12	1.34 ± 0.12	1.11 ± 0.11	0.97 ± 0.05
	X4	0.26 ± 0.04	0.25 ± 0.01	0.25 ± 0.01	0.22 ± 0.01	0.2 ± 0.02	0.15 ± 0.02	0.11 ± 0.02	0.09 ± 0.01
	X5	0.03 ± 0.01	0.03 ± 0.01	0.03 ± 0.01	0.02 ± 0.01	0.02 ± 0.01	0.02 ± 0.01	0.01 ± 0.01	0.01 ± 0.01
pH 7.0	X1	0.1 ± 0.01	0.17 ± 0.01	0.25 ± 0.01	0.3 ± 0.01	0.4 ± 0.04	0.6 ± 0.04	0.93 ± 0.05	1.23 ± 0.06
	X2	0.76 ± 0.06	0.8 ± 0.03	0.77 ± 0.02	0.78 ± 0.02	0.78 ± 0.04	0.78 ± 0.04	0.78 ± 0.04	0.75 ± 0.05
	X3	1.33 ± 0.1	1.29 ± 0.06	1.25 ± 0.05	1.22 ± 0.04	1.22 ± 0.07	1.14 ± 0.06	1.07 ± 0.06	0.95 ± 0.05
	X4	0.3 ± 0.04	0.27 ± 0.01	0.26 ± 0.02	0.25 ± 0.02	0.24 ± 0.02	0.2 ± 0.02	0.17 ± 0.01	0.13 ± 0.02
	X5	0.03 ± 0.01	0.03 ± 0.01	0.03 ± 0.01	0.03 ± 0.01	0.03 ± 0.01	0.02 ± 0.01	0.03 ± 0.01	0.03 ± 0.02

Table S3. Raw data with standard deviations for figure **4B**

1440 min	X1 (mg/ml)	X2 (mg/ml)	X3 (mg/ml)	X4 (mg/ml)	X5 (mg/ml)
33°C	1.41 ± 0.17	0.5 ± 0.07	0.57 ± 0.09	0.11 ± 0.03	0.01 ± 0.01
37°C	1.94 ± 0.18	0.52 ± 0.05	0.56 ± 0.05	0.1 ± 0.01	0.01 ± 0.01
41°C	2.09 ± 0.14	0.38 ± 0.04	0.42 ± 0.04	0.08 ± 0.01	0.01 ± 0.01
45°C	2.13 ± 0.06	0.34 ± 0.05	0.35 ± 0.03	0.11 ± 0.03	0.04 ± 0.01
49°C	2.02 ± 0.17	0.26 ± 0.07	0.32 ± 0.09	0.11 ± 0.04	0.05 ± 0.02

2440 min	X1 (mg/ml)	X2 (mg/ml)	X3 (mg/ml)	X4 (mg/ml)	X5 (mg/ml)
33°C	1.89 ± 0.1	0.27 ± 0.02	0.24 ± 0.02	0.03 ± 0.01	0.01 ± 0.01
37°C	2.49 ± 0.07	0.22 ± 0.04	0.19 ± 0.04	0.02 ± 0.01	0.01 ± 0.01
41°C	2.58 ± 0.03	0.11 ± 0.03	0.1 ± 0.03	0.02 ± 0.01	0.01 ± 0.01
45°C	2.69 ± 0.17	0.1 ± 0.02	0.1 ± 0.02	0.02 ± 0.01	0.01 ± 0.02
49°C	2.58 ± 0.16	0.07 ± 0.02	0.07 ± 0.01	0.02 ± 0.01	0.01 ± 0.01

Table S4. Raw data with standard deviations for figure **4C**

	X1 (mg/ml)	X2 (mg/ml)	X3 (mg/ml)	X4 (mg/ml)	X5 (mg/ml)
soluble	2 ± 0.17	0.65 ± 0.03	0.44 ± 0.02	0.04 ± 0.01	0 ± 0
fibril 0 rpm	1.94 ± 0.18	0.52 ± 0.05	0.56 ± 0.05	0.1 ± 0.01	0.01 ± 0.01
fibril 500 rpm	2.09 ± 0.13	0.6 ± 0.04	0.5 ± 0.03	0.05 ± 0.01	0.01 ± 0.01
fibril 1000 rpm	2.44 ± 0.06	0.34 ± 0.03	0.29 ± 0.03	0.03 ± 0.01	0.01 ± 0.01

Table S5. Raw data with standard deviations for figure **5A**

		X1 (mg/ml)	X2 (mg/ml)	X3 (mg/ml)	X4 (mg/ml)	X5 (mg/ml)
Round 1	33°C	1.41 ± 0.05	0.54 ± 0.04	0.68 ± 0.05	0.34 ± 0.02	0.03 ± 0.01
	37°C	1.69 ± 0.06	0.49 ± 0.03	0.59 ± 0.04	0.27 ± 0.01	0.03 ± 0.01
	41°C	1.95 ± 0.14	0.39 ± 0.04	0.5 ± 0.07	0.27 ± 0.03	0.04 ± 0.01
	45°C	2.04 ± 0.13	0.31 ± 0.03	0.39 ± 0.05	0.25 ± 0.02	0.05 ± 0.01
	49°C	2.08 ± 0.2	0.26 ± 0.03	0.3 ± 0.05	0.2 ± 0.02	0.06 ± 0.02
Round 2	33°C	1.33 ± 0.07	0.53 ± 0.01	0.62 ± 0.04	0.26 ± 0.02	0.02 ± 0.01
	37°C	1.65 ± 0.13	0.49 ± 0.03	0.57 ± 0.03	0.24 ± 0.01	0.01 ± 0.01
	41°C	1.51 ± 0.12	0.33 ± 0.01	0.54 ± 0.02	0.43 ± 0.02	0.14 ± 0.01
	45°C	1.02 ± 0.06	0.15 ± 0.02	0.3 ± 0.02	0.38 ± 0.02	0.25 ± 0.02
	49°C	0.55 ± 0.03	0.06 ± 0.01	0.12 ± 0.01	0.16 ± 0.02	0.13 ± 0.01
Round 3	33°C	1.27 ± 0.1	0.51 ± 0.03	0.65 ± 0.03	0.33 ± 0.02	0.03 ± 0.02
	37°C	1.79 ± 0.1	0.48 ± 0.03	0.54 ± 0.09	0.29 ± 0.03	0.03 ± 0.02
	41°C	1.17 ± 0.15	0.22 ± 0.06	0.41 ± 0.11	0.4 ± 0.07	0.25 ± 0.06
	45°C	0.83 ± 0.08	0.11 ± 0.02	0.22 ± 0.03	0.27 ± 0.02	0.19 ± 0.02
	49°C	0.32 ± 0.05	0.04 ± 0.01	0.07 ± 0.02	0.09 ± 0.01	0.07 ± 0.02
Round 4	33°C	1.24 ± 0.01	0.46 ± 0.06	0.57 ± 0.09	0.3 ± 0.04	0.04 ± 0.01
	37°C	1.54 ± 0.05	0.41 ± 0.03	0.51 ± 0.06	0.32 ± 0.03	0.05 ± 0.01
	41°C	1.08 ± 0.07	0.2 ± 0.05	0.4 ± 0.09	0.43 ± 0.07	0.23 ± 0.04
	45°C	0.69 ± 0.02	0.09 ± 0.01	0.18 ± 0.02	0.22 ± 0.01	0.17 ± 0.01
	49°C	0.26 ± 0.03	0.03 ± 0.01	0.06 ± 0.01	0.09 ± 0.01	0.07 ± 0.01
Round 5	33°C	1.31 ± 0.05	0.46 ± 0.04	0.58 ± 0.06	0.28 ± 0.02	0.03 ± 0.01
	37°C	1.55 ± 0.07	0.38 ± 0.05	0.58 ± 0.07	0.38 ± 0.03	0.07 ± 0.01
	41°C	1.09 ± 0.06	0.17 ± 0.01	0.39 ± 0.02	0.41 ± 0.02	0.22 ± 0.02
	45°C	0.66 ± 0.04	0.07 ± 0.01	0.13 ± 0.02	0.18 ± 0.02	0.11 ± 0.01
	49°C	0.22 ± 0.04	0.02 ± 0.01	0.05 ± 0.01	0.07 ± 0.01	0.04 ± 0.01
Round 6	33°C	1.25 ± 0.24	0.44 ± 0.02	0.55 ± 0.02	0.33 ± 0.02	0.05 ± 0.01
	37°C	1.24 ± 0.04	0.33 ± 0.02	0.56 ± 0.01	0.47 ± 0.03	0.14 ± 0.03
	41°C	1.02 ± 0.07	0.15 ± 0.01	0.33 ± 0.04	0.41 ± 0.02	0.24 ± 0.02
	45°C	0.64 ± 0.01	0.06 ± 0.01	0.12 ± 0.02	0.16 ± 0.01	0.11 ± 0.01
	49°C	0.2 ± 0.02	0.02 ± 0.01	0.06 ± 0.01	0.07 ± 0.01	0.04 ± 0.01
Round 7	33°C	1.33 ± 0.05	0.41 ± 0.04	0.58 ± 0.04	0.33 ± 0.02	0.05 ± 0.01
	37°C	1.33 ± 0.05	0.34 ± 0.01	0.57 ± 0.03	0.46 ± 0.02	0.14 ± 0.01
	41°C	1 ± 0.1	0.1 ± 0.09	0.32 ± 0.03	0.39 ± 0.01	0.23 ± 0.01
	45°C	0.59 ± 0.03	0.05 ± 0.01	0.12 ± 0.01	0.14 ± 0.02	0.1 ± 0.01
	49°C	0.15 ± 0.03	0.02 ± 0.01	0.05 ± 0.02	0.08 ± 0.01	0.04 ± 0.01
Round 8	33°C	1.18 ± 0.02	0.43 ± 0.02	0.53 ± 0.04	0.33 ± 0.02	0.06 ± 0.01
	37°C	1.13 ± 0.08	0.26 ± 0.02	0.48 ± 0.05	0.48 ± 0.02	0.19 ± 0.02
	41°C	0.88 ± 0.08	0.13 ± 0.01	0.29 ± 0.01	0.34 ± 0.01	0.21 ± 0.01
	45°C	0.52 ± 0.01	0.05 ± 0.01	0.1 ± 0.01	0.13 ± 0.01	0.1 ± 0.01
	49°C	0.11 ± 0.03	0.02 ± 0.01	0.04 ± 0.01	0.05 ± 0.01	0.04 ± 0.01

Table S6. Raw data with standard deviations for figure **5B**

		X1 (mg/ml)	X2 (mg/ml)	X3 (mg/ml)	X4 (mg/ml)	X5 (mg/ml)
10 min	0 rpm	0 ± 0	0.04 ± 0.01	0.18 ± 0.03	0.15 ± 0.03	0.14 ± 0.03
	500 rpm	0.02 ± 0.01	0.05 ± 0.01	0.23 ± 0.03	0.2 ± 0.02	0.14 ± 0.01
	1000rpm	0.02 ± 0.01	0.05 ± 0.01	0.22 ± 0.04	0.2 ± 0.05	0.16 ± 0.04
60 min	0 rpm	0.15 ± 0.03	0.2 ± 0.01	0.66 ± 0.04	0.43 ± 0.03	0.25 ± 0.02
	500 rpm	0.08 ± 0.01	0.26 ± 0.03	0.81 ± 0.06	0.48 ± 0.05	0.19 ± 0.02
	1000rpm	0.12 ± 0.01	0.26 ± 0.01	0.85 ± 0.05	0.52 ± 0.05	0.24 ± 0.03
120 min	0 rpm	0.25 ± 0.06	0.33 ± 0.04	0.88 ± 0.04	0.5 ± 0.01	0.22 ± 0.02
	500 rpm	0.2 ± 0.01	0.45 ± 0.02	1.04 ± 0.05	0.5 ± 0.03	0.12 ± 0.02
	1000rpm	0.31 ± 0.02	0.43 ± 0.03	1.07 ± 0.06	0.52 ± 0.02	0.14 ± 0.02
240 min	0 rpm	0.51 ± 0.01	0.53 ± 0.02	1.08 ± 0.04	0.5 ± 0.02	0.15 ± 0.02
	500 rpm	0.46 ± 0.07	0.68 ± 0.02	1.17 ± 0.04	0.44 ± 0.02	0.06 ± 0.01
	1000rpm	0.64 ± 0.08	0.65 ± 0.08	1.17 ± 0.12	0.44 ± 0.05	0.07 ± 0.01
4800 min	0 rpm	0.84 ± 0.07	0.63 ± 0.06	0.98 ± 0.07	0.35 ± 0.02	0.06 ± 0.02
	500 rpm	1.04 ± 0.06	0.93 ± 0.02	1.2 ± 0.03	0.3 ± 0.02	0.03 ± 0.01
	1000rpm	1.16 ± 0.17	0.74 ± 0.12	0.99 ± 0.15	0.24 ± 0.05	0.03 ± 0.01
7200 min	0 rpm	1.11 ± 0.35	0.58 ± 0.19	0.78 ± 0.24	0.22 ± 0.06	0.03 ± 0.01
	500 rpm	1.18 ± 0.15	0.7 ± 0.1	0.81 ± 0.09	0.15 ± 0.02	0.02 ± 0.01
	1000rpm	1.52 ± 0.12	0.66 ± 0.05	0.74 ± 0.05	0.13 ± 0.02	0.01 ± 0.01
960 min	0 rpm	1.46 ± 0.32	0.61 ± 0.13	0.74 ± 0.15	0.17 ± 0.04	0.02 ± 0.01
	500 rpm	1.65 ± 0.11	0.73 ± 0.03	0.71 ± 0.05	0.08 ± 0.01	0.01 ± 0.01
	1000rpm	1.97 ± 0.1	0.49 ± 0.03	0.46 ± 0.03	0.05 ± 0.01	0.01 ± 0.01
1200 min	0 rpm	1.72 ± 0.2	0.63 ± 0.08	0.7 ± 0.09	0.14 ± 0.03	0.02 ± 0.01
	500 rpm	1.76 ± 0.09	0.62 ± 0.04	0.55 ± 0.04	0.06 ± 0.01	0.01 ± 0.01
	1000rpm	2.44 ± 0.07	0.39 ± 0.03	0.33 ± 0.02	0.03 ± 0.01	0.01 ± 0.01
1440 min	0 rpm	1.94 ± 0.18	0.52 ± 0.05	0.56 ± 0.05	0.1 ± 0.01	0.01 ± 0.01
	500 rpm	2.09 ± 0.13	0.6 ± 0.04	0.5 ± 0.03	0.05 ± 0.01	0.01 ± 0.01
	1000rpm	2.44 ± 0.06	0.34 ± 0.03	0.29 ± 0.03	0.03 ± 0.01	0.01 ± 0.01

Table S7. Raw data with standard deviations for figure **6D**

Buffer	pH	µM/min
KPi	6	0.08 ± 0.01
KPi	6.5	0.36 ± 0.01
KPi	7	1.21 ± 0.07
KPi	7.5	5.13 ± 0.19
KPi	8	15.43 ± 0.7
KPi Tris	8.5	23.61 ± 0.81
KPi Tris	9	24.26 ± 0.9
Tris	7.5	24.09 ± 0.28
Tris	8	27.32 ± 0.31
Tris	8.5	28.36 ± 0.64
Tris	9	25.31 ± 0.5
Gly-NaOH	9.5	21.31 ± 0.26

Table S8. Raw data with standard deviations for figure **6E**

	X1 (mg/ml)	X2 (mg/ml)	X3 (mg/ml)	X4 (mg/ml)	X5 (mg/ml)
RT, pH7.5	0.17 ± 0.02	0.42 ± 0.03	0.77 ± 0.07	0.31 ± 0.03	0.08 ± 0.1
RT, pH6	0.73 ± 0.06	0.52 ± 0.05	0.66 ± 0.06	0.17 ± 0.02	0.03 ± 0.11
37, pH6	1.94 ± 0.18	0.52 ± 0.05	0.56 ± 0.05	0.1 ± 0.01	0.01 ± 0.01

Table S9. Raw data with standard deviations for figure **7A**

t(h)	X1 (mg/ml)	X2 (mg/ml)	X3 (mg/ml)	XL (mg/ml)
0	0.02 ± 0.01	0.05 ± 0.01	0.21 ± 0.02	0 ± 0
24	0.23 ± 0.03	0.43 ± 0.04	0.7 ± 0.03	0 ± 0
48	0.52 ± 0.03	0.48 ± 0.02	0.59 ± 0.03	0.03 ± 0.01
72	0.9 ± 0.03	0.46 ± 0.05	0.49 ± 0.07	0.17 ± 0.01
96	1.14 ± 0.08	0.31 ± 0.02	0.29 ± 0.02	0.22 ± 0.03
120	1.48 ± 0.08	0.25 ± 0.05	0.22 ± 0.03	0.3 ± 0.03

Table S10. Raw data with standard deviations for figure **7B**

t(h)	X1 (mg/ml)	X2 (mg/ml)	X3 (mg/ml)	XL (mg/ml)	X2L (mg/ml)	X3L (mg/ml)
0	0 ± 0	0.04 ± 0.01	0.2 ± 0.04	0 ± 0	0.02 ± 0.01	0.02 ± 0.01
24	0 ± 0	0.4 ± 0.04	0.72 ± 0.06	0 ± 0	0.1 ± 0.03	0.32 ± 0.11
48	0.01 ± 0.01	0.16 ± 0.03	0.23 ± 0.04	0.01 ± 0.01	0.39 ± 0.05	0.73 ± 0.09
72	0.04 ± 0.01	0.15 ± 0.01	0.15 ± 0.01	0.01 ± 0.01	0.62 ± 0.05	1.17 ± 0.07
96	0.03 ± 0.01	0.12 ± 0.01	0.12 ± 0.02	0.01 ± 0.01	0.81 ± 0.02	1.47 ± 0.03
120	0.02 ± 0.01	0.06 ± 0.01	0.06 ± 0.01	0.01 ± 0.01	0.81 ± 0.03	1.43 ± 0.02

Table S11. Raw data with standard deviations for figure **7C**

t(h)	X1 (mg/ml)	XL (mg/ml)
0	1.92 ± 0.02	0 ± 0
24	1.28 ± 0.04	0.19 ± 0.03
48	1.19 ± 0.06	0.24 ± 0.04
72	1.17 ± 0.05	0.28 ± 0.05
96	1.28 ± 0.1	0.36 ± 0.04
120	1.13 ± 0.01	0.33 ± 0.04

Table S12. Raw data with standard deviations for figure **7D**

t(h)	X1 (mg/ml)	XL (mg/ml)
0	1.92 ± 0.02	0 ± 0
1	1.78 ± 0.02	0.12 ± 0.01
5	1.35 ± 0.07	0.6 ± 0.05
10	0.81 ± 0.04	1.15 ± 0.06
16	0.29 ± 0.02	1.41 ± 0.02
24	0.07 ± 0.01	1.85 ± 0.11

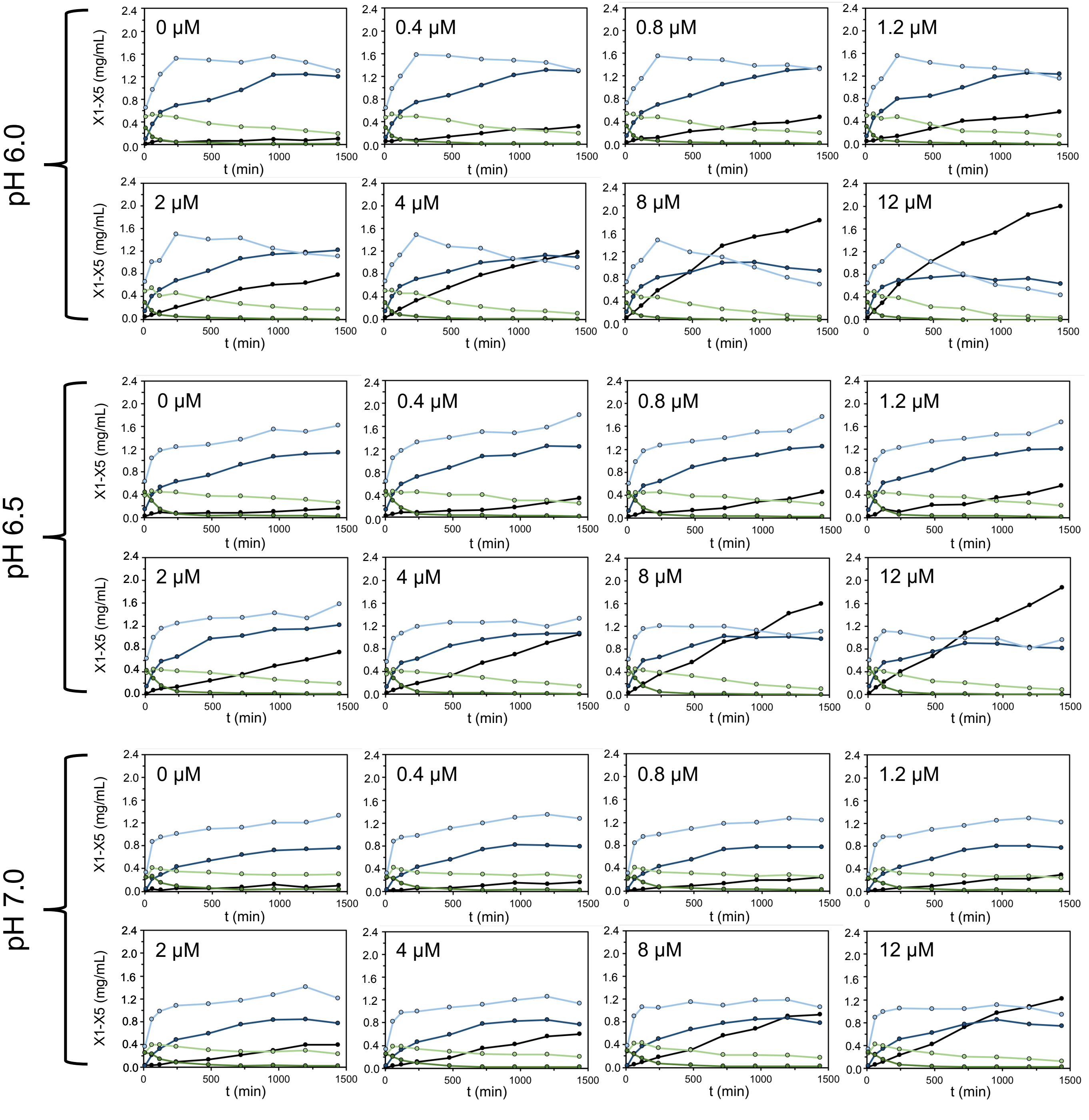

Figure S1. Time dependent accumulation of xylose (black), xylobiose (dark blue), xylotriose (light blue), xylotetraose (light green), and xylopentaose (dark green). Xylose (X1) and the xylooligos (XOS or X2-X5) are liberated by hydrolysis of beechwood xylan with an enzyme cocktail containing soluble Xylanase A (XynA) from *Bacillus subtillis* and GH39 β-Xylosidase II (βXyl) from *Caulobacter crescentus*. The XynA concentration was constant at 0.4 µM, the concentration of βXyl was varied between 0-12 µM (indicated in the top left corner of each plot). The biocatalytic reaction was carried out at 37°C and pH 6.0-7.0.

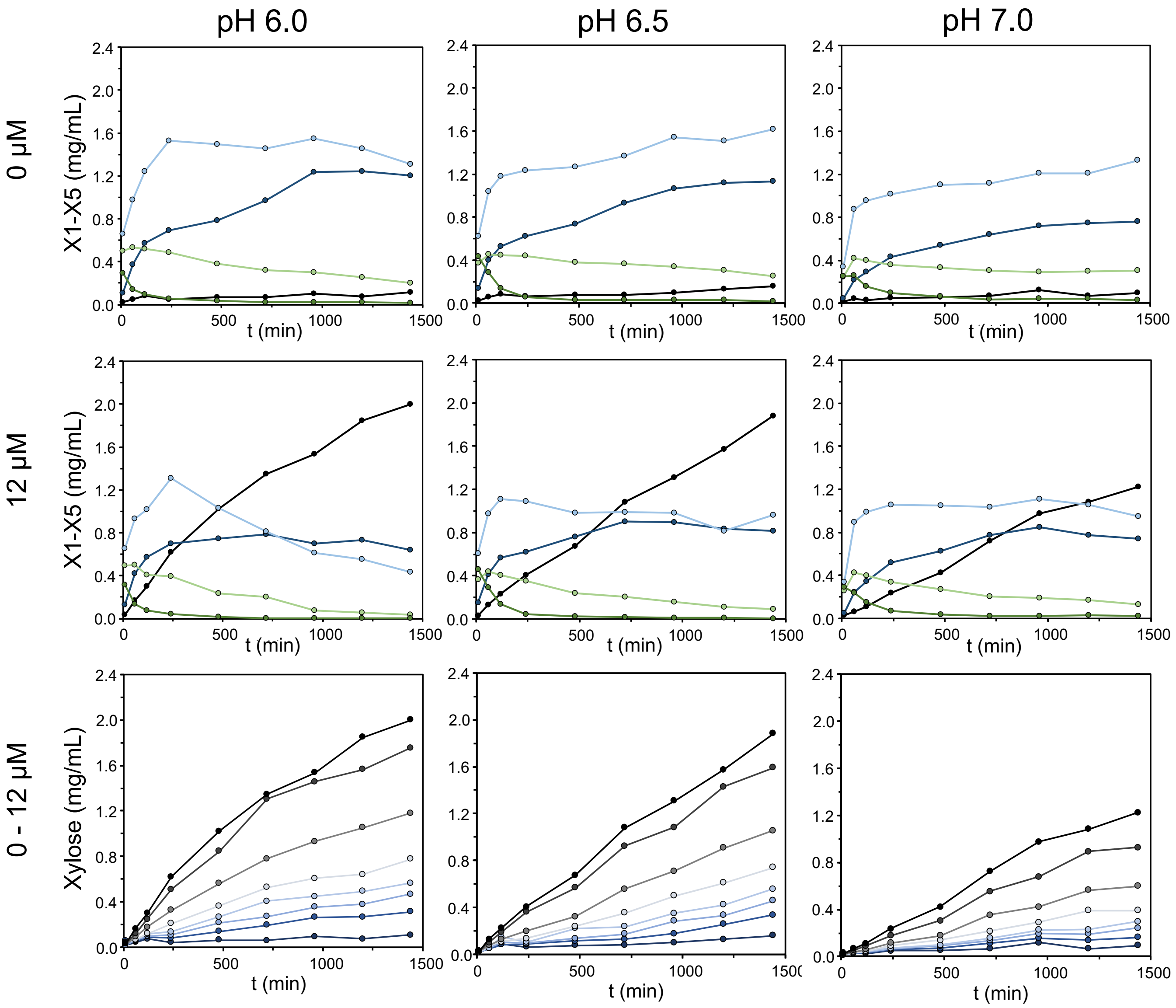

Figure S2. Summary of **Figure S1**. The label on the y-axis indicates the βXyl concentration in the enzyme cocktail, in addition to 0.4 µM XynA. The bottom row is a compilation of plots that only contain the time-dependent accumulation of xylose. The lines are colored using a gradient from black to dark blue, which indicates the βXyl concentration (dark blue 0 µM to black 12 µM).

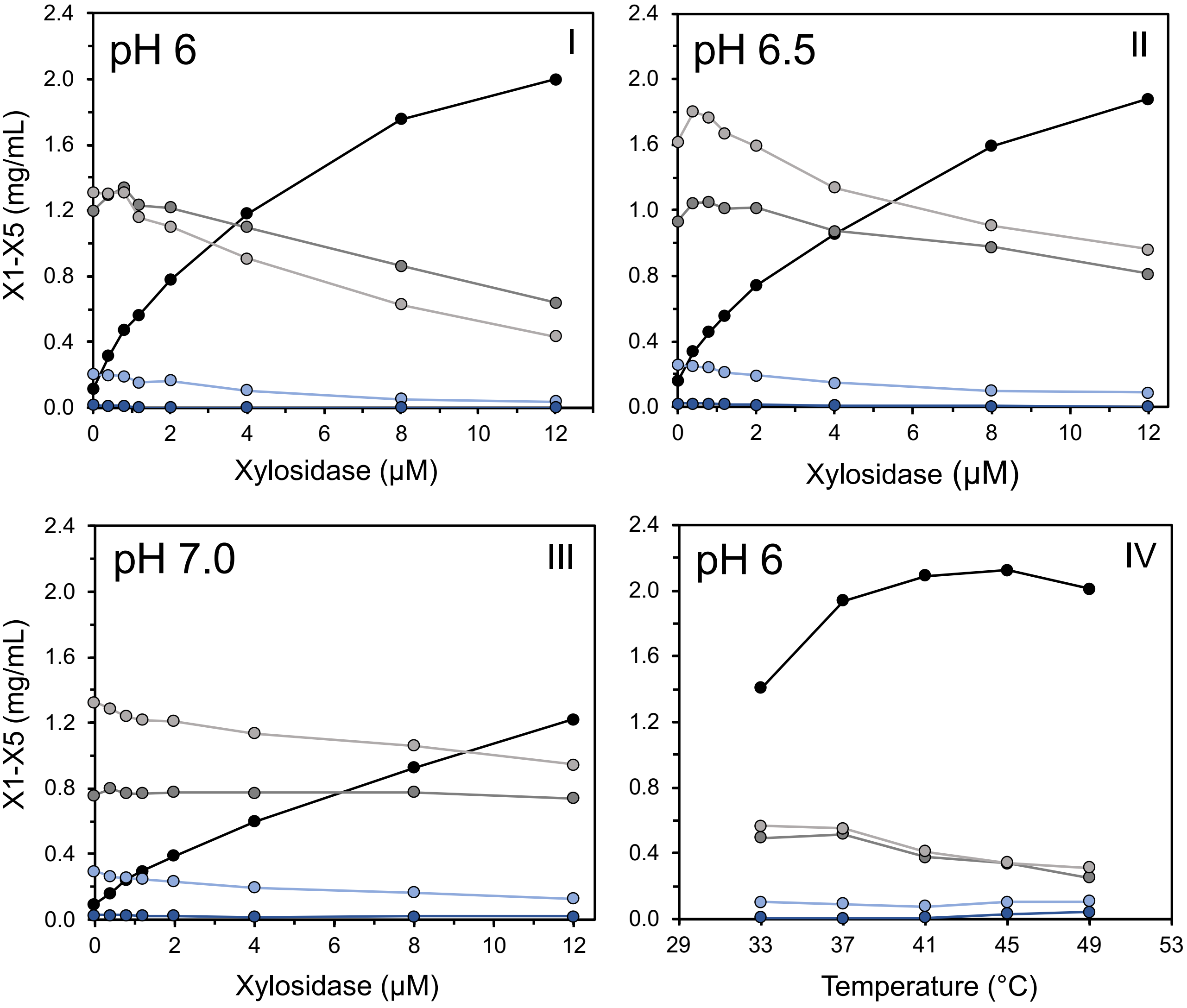

Figure S3. The yield of xylose and XOS as a function of βXyl concentration (plot I-III) and temperature (plot IV) after incubation for 24 h. The plots I-III are a different representation of **Figure 4A** in the paper and summarize the concentration of X1-X5 as a function of βXyl concentration after 24 h incubation at 37°C (black - X1, dark grey - X2, light grey - X3, light blue - X4, dark blue - X5). In plot IV, the yield of X1-5 (coloring as in plot I-III) is shown as a function of temperature. Instead of soluble enzymes, the biocatalysts were displayed on Sup35(1-61) protein nanofibrils (PNF). The enzyme concentration in this assay was 0.4 µM XynA and 12 µM βXyl.

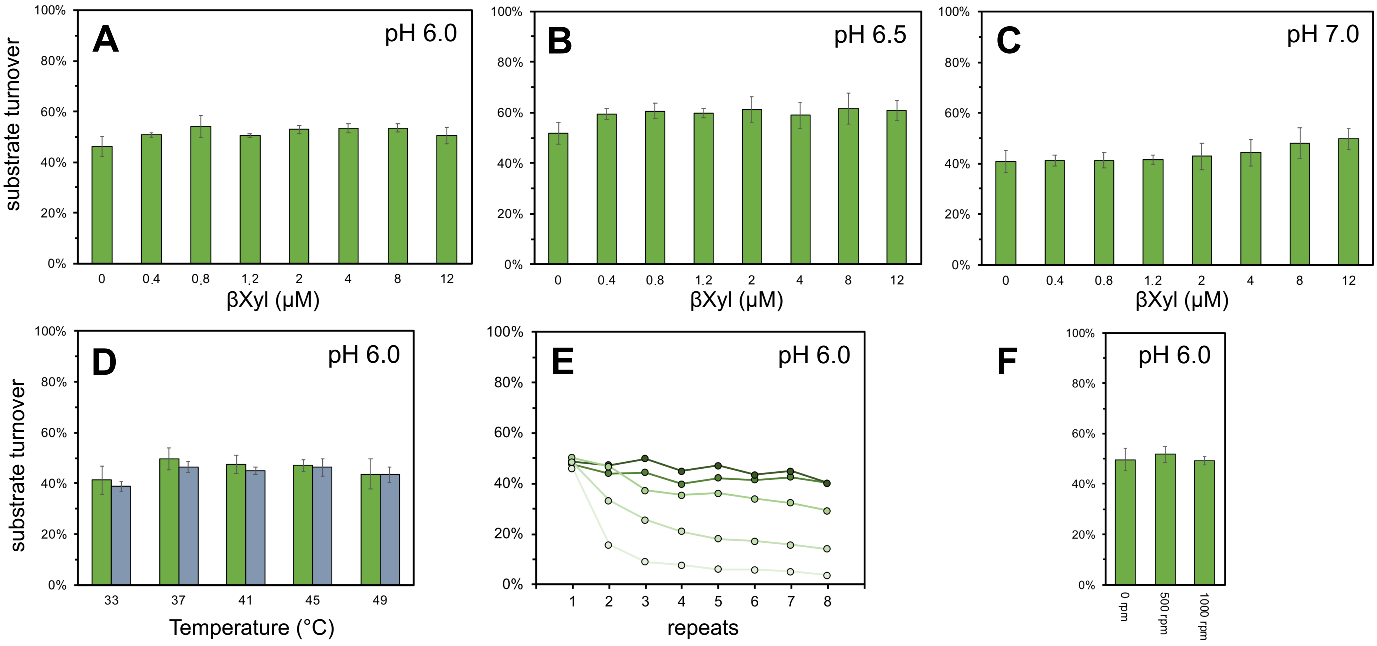

Figure S4. Summary of the substrate (beechwood xylan) turnover using a XynA/βXyl enzyme cocktail. The maximal xylose concentration of the substrate solution (1% beechwood xylan) was determined by acid hydrolysis (see material and methods). Then, the substrate turnover in % was calculated as the sum of X1-X5 divided by the maximal measured xylose concentration. (A-C) Substrate turnover after 24 h and 37°C using pH 6.0, 6.5 and 7.0 using soluble enzymes. These values are a complement to **Figure S1** were the time dependent accumulation of X1-X5 was studied as a function of βXyl concentration (0-12 μM) and constant XynA (0.4 μM). (D-F) The substrate was hydrolyzed with enzymatic functionalized nanofibrils. (D) after 24 h (green) or after 48 h (grey) as a function of temperature (0.4 μM XynA, 12 μM βXyl). (E) The assay was carried out as in (D), but the fibrils were reused eight times. One repeat refers to a 24 h incubation. Each line represents a temperature (33, 37, 41, 45 or 49 °C), which were colored from dark green to light green using the same order. (F) The enzymatic functionalized fibrils (0.4 μM XynA, 12 μM βXyl) were incubated at 37°C and agitated at 0, 500 or 1000 rpm.

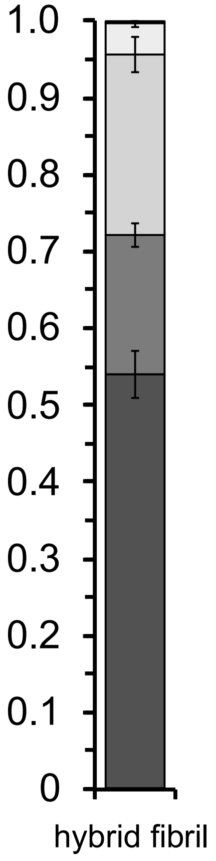

Figure S5. Relative XOS yield after the hydrolysis of beechwood xylan using hybrid fibrils. This experiment was performed to verify if the xylose yield could be increased using hybrid fibrils, compared to the mixed fibril setup in **Figure 4C**. The rationale of this experiment was that substrate channeling from XynA to βXyl could accelerate the hydrolysis, if both enzymes are located in close proximity on the same fibril. The hybrid fibrils were obtained by fibrillating the chimeric *C*XynA simultaneously with *C*βXyl in the presence of Sup35. The fibrils had a doping frequency of 1:0.33 with respect to Sup35 over the chimeric proteins. The assay conditions were identical to the setup in **Figure 4C**. The final enzyme concentration in the hydrolysis reaction was 0.4 µM XynA and 12 µM βXyl, at 37 °C and pH 6.0. The error bars indicate the standard deviation of three experimental replicates. This experiment shows that the use of hybrid fibrils for beechwood xylan hydrolysis is not favorable with respect to the xylose yield. Here, only 55% xylose was obtained after a 24h incubation period, compared to 65% using the mixed fibril enzyme cocktail (**Figure 4C**).

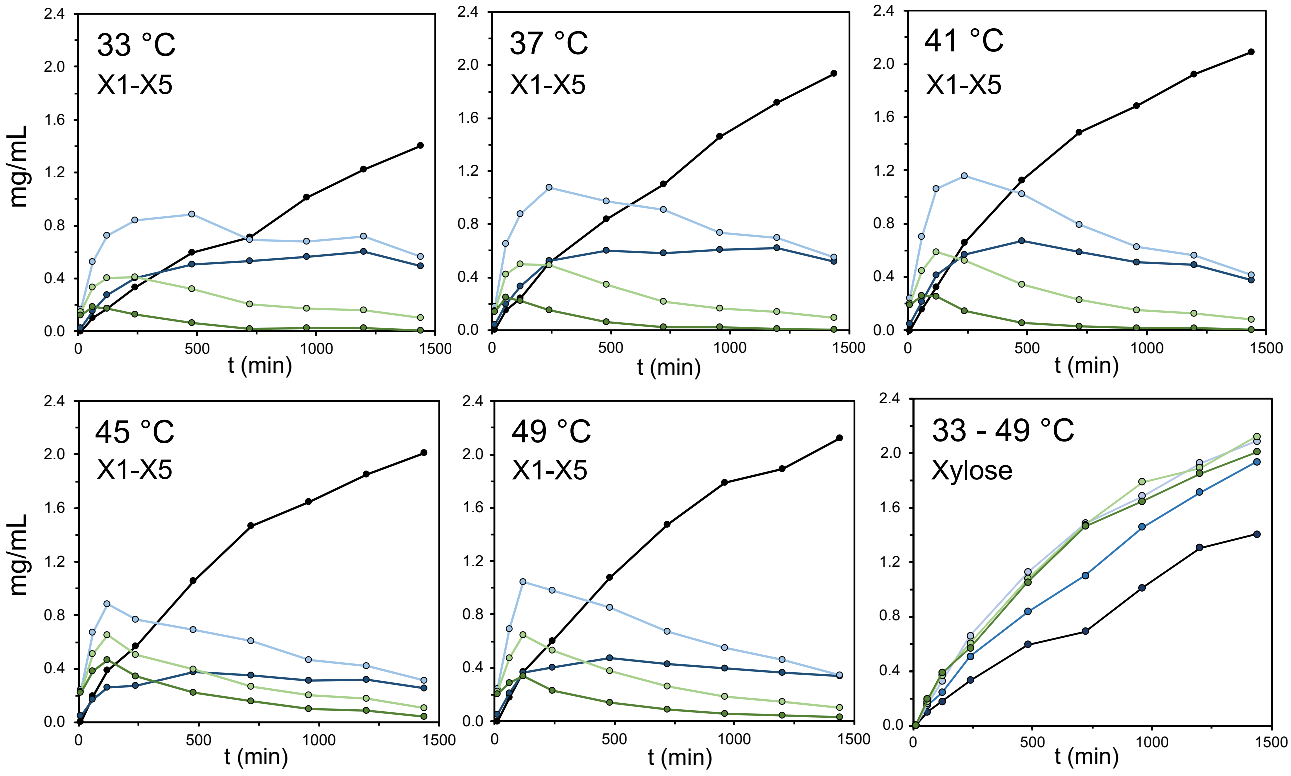

Figure S6. The effect of the temperature on the time-dependent yield of xylose and the XOS. These plots are a complement to **Figure S3**, plot IV and **Figure S4F**. The colored lines refer to xylose (black), xylobiose (dark blue), xylotriose (light blue), xylotetraose (light green), and xylopentaose (dark green) in all plots designated 'X1-X5'. In the plot designated 'Xylose' the lines for the accumulation of xylose are merged in one plot. Here, the colors refer to 33°C (black), 37°C (dark blue), 41°C (light blue), 45°C (light green), and 49°C (dark green).

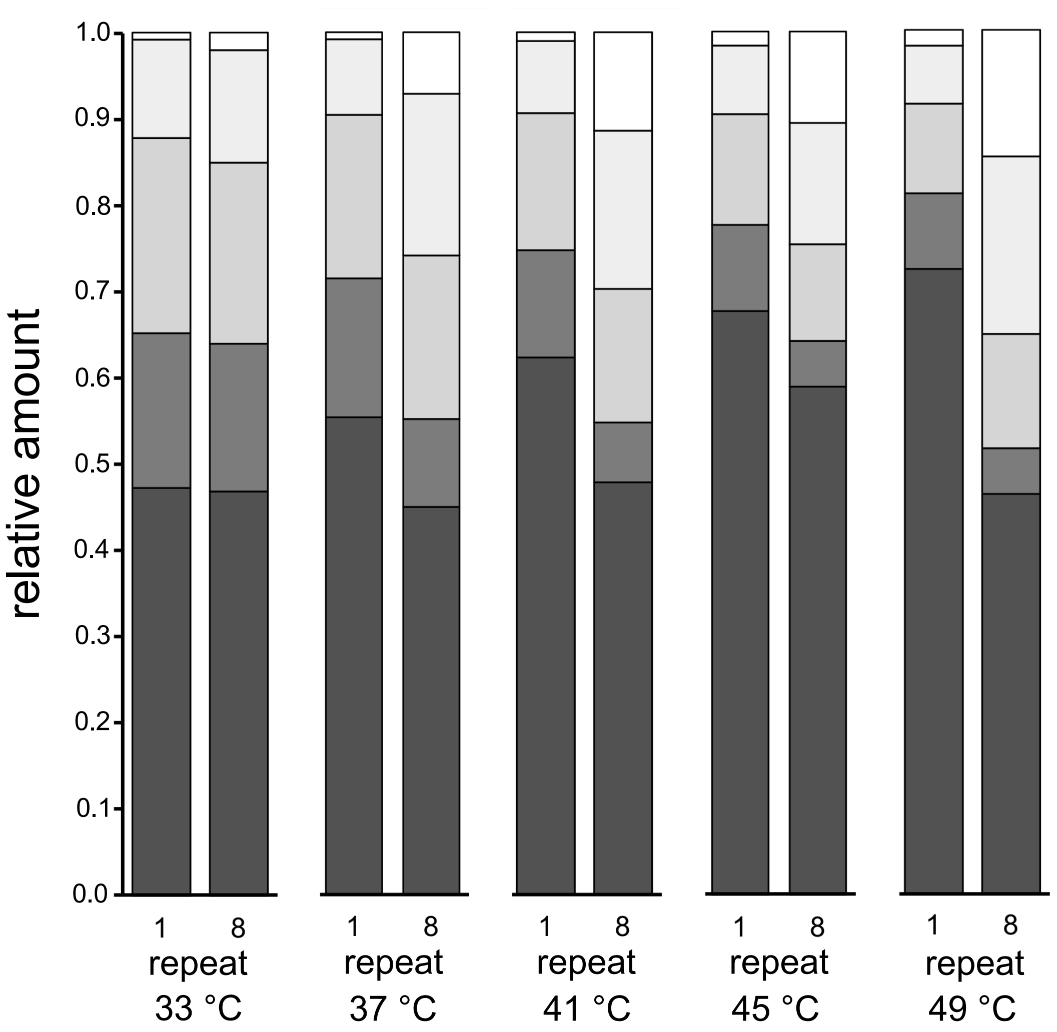

Figure S7. The relative amount of X1-X5 (dark grey - X1 - to white - X5) are compared after one and eight fibril reuses at 33-49 °C. This figure complements **Figure 5** in the paper.

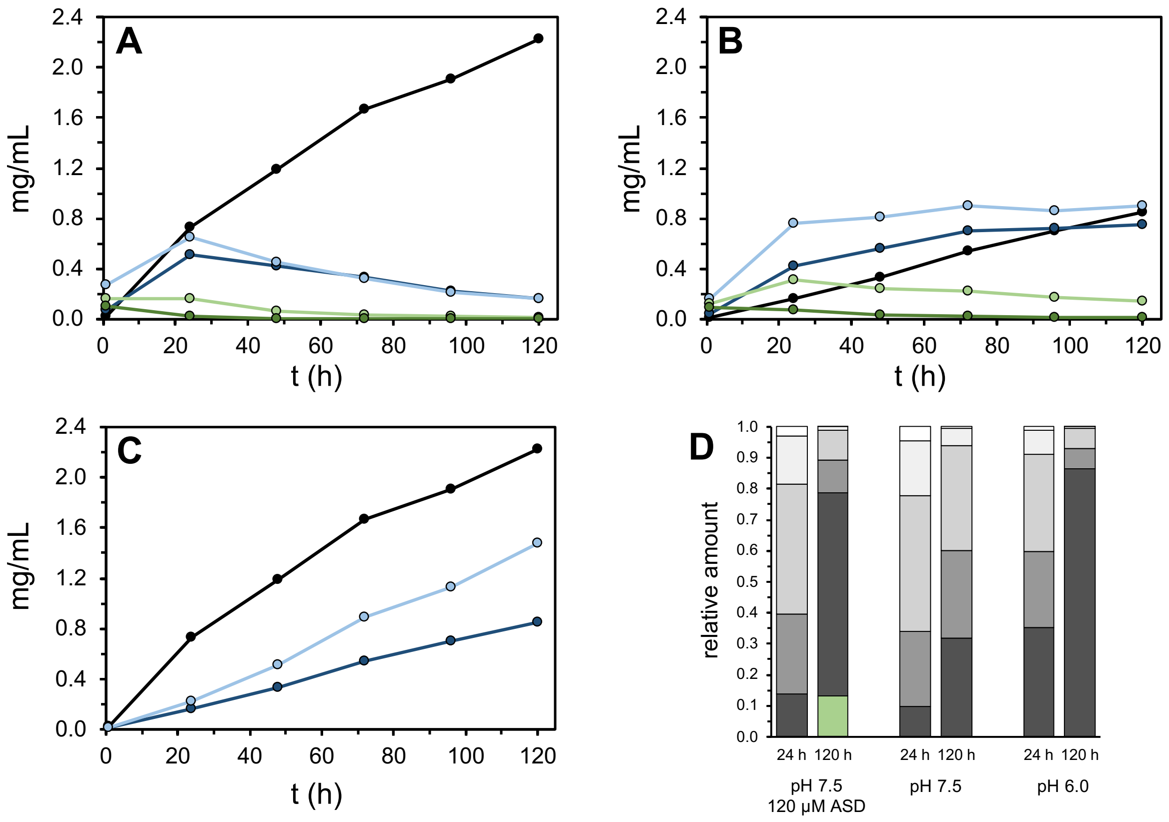

Figure S8. Liberation of X1-X5 from 1% beechwood xylan using conditions that are more favorable for PNF functionalized with aldose sugar dehydrogenase (ASD) from *Escherichia coli*. (A) The XynA/βXyl (0.4 µM/12 µM) enzyme cocktail is incubated with beechwood xylan at pH 6.0 and room temperature (xylose - black, xylobiose - dark blue, xylotriose - light blue, xylotetraose - light green, and xylopentaose - dark green). (B) Identical setup as (A) but at pH 7.5. (C) Xylose concentration over time are merged in one plot. Black: xylose accumulation from (A); Dark Blue: xylose accumulation from (B); Light blue: xylose accumulation from **Figure 7A**. In this case the conditions were identical to (B), but in the presence of ASD (120 µM) in a sealed reaction container. (D) The relative amount after 24/120 h of X1-X5 (colored in the same order from dark grey to white) and xylonolactone (green). The figure is a complement comparison of plot A (pH 6.0), plot B (pH 7.5) in combination with **Figure 7A** (pH 7.5, 120 µM ASD).

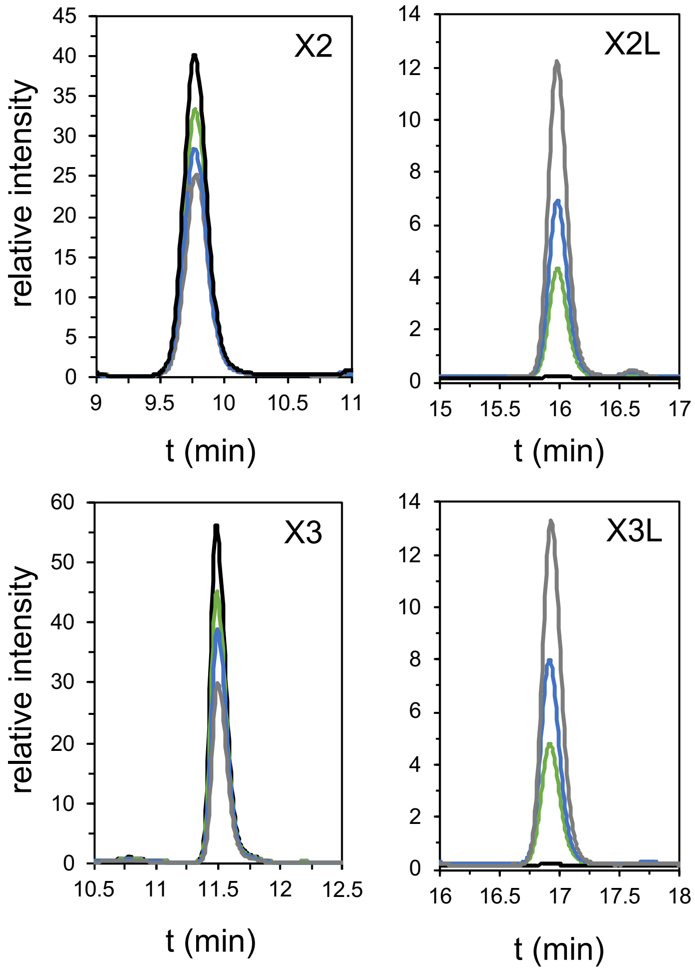

Figure S9. Oxidation of xylobiose (X2) and xylotriose (X3) into xylobionolactone (X2L) and xylotrionolactone (X3L), respectively. Known amounts of X2 and X3 were oxidized with soluble ASD (34 µM). The relative intensities of the peaks in the HPAEC chromatogram after 10 min (black), 60 min (green), 120 min (blue), and 240 min (grey) are shown. To be able to quantify X2L and X3L, we integrated the peaks and assumed that the time dependent amount produced is equal to the decrease of X2 and X3, respectively.

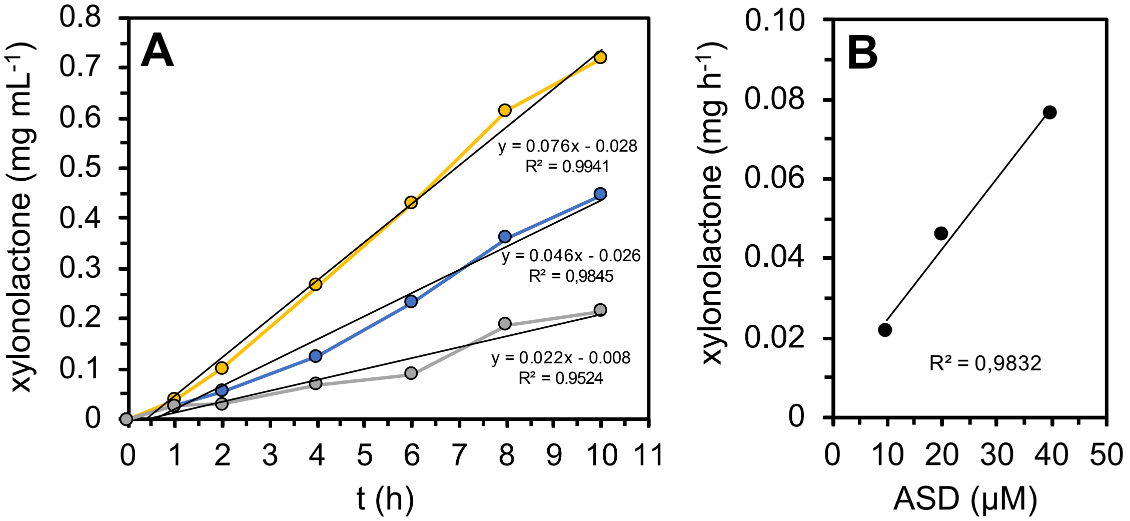

Figure S10. Oxidation of xylose and synthesis of xylonolactone using different concentrations of ASD functionalized Sup35 nanofibrils. (A) ASD fibrils 10 µM (grey), 20 µM (blue) and 40 µM (yellow) were incubated at room temperature with hydrolyzed beechwood xylan, after the XynA/βXyl (0.4 µM/12 µM) fibrils were removed and the pH was adjusted to 9.0. (A & B) The production of xylonolactone is linear with respect to time and ASD concentration.

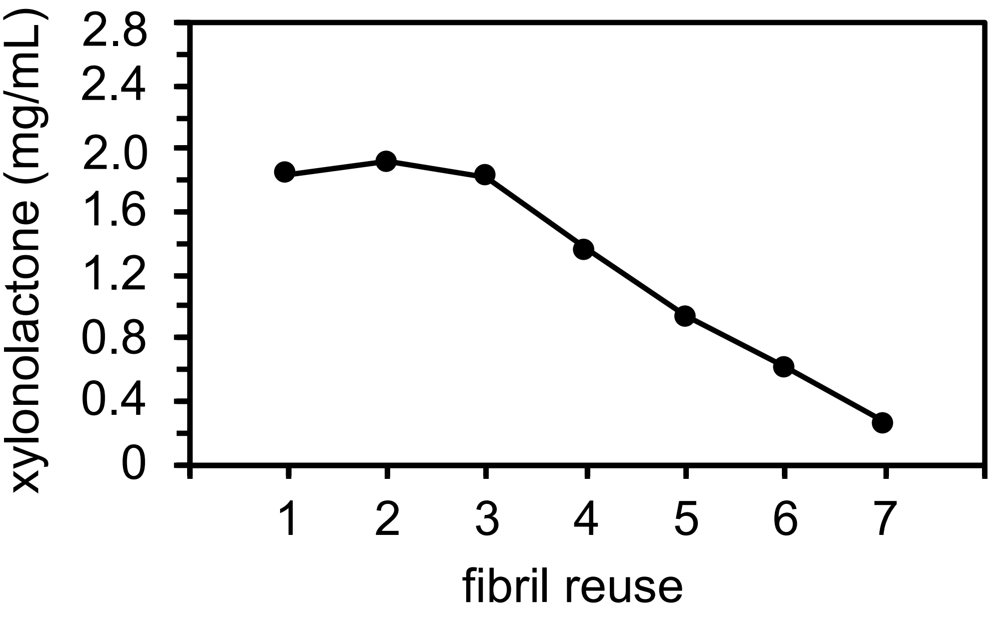

Figure S11. Reuse of ASD functionalized fibrils (40 µM enzyme) for the oxidation of xylose (~2 mg/mL) to xylonolactone. The fibrils were incubated together with the substrate at pH 9.0 and RT in a reaction container that has three holes to ensure continuous oxygenation. After each cycle, the fibrils were pelleted, washed and resuspended in fresh substrate solution.
